# Supercritical CO_2_ Extraction of Palladium Oxide from an Aluminosilicate-Supported Catalyst Enhanced by a Combination of Complexing Polymers and Piperidine

**DOI:** 10.3390/molecules26030684

**Published:** 2021-01-28

**Authors:** Andrea Ruiu, Bernhard Bauer-Siebenlist, Marin Senila, W. S. Jennifer Li, Karine Seaudeau-Pirouley, Patrick Lacroix-Desmazes, Thorsten Jänisch

**Affiliations:** 1 ICGM, Univ Montpellier, CNRS, ENSCM, 34095 Montpellier, France; andrea1.ruiu@gmail.com (A.R.); wing-sze.li@enscm.fr (W.S.J.L.); 2 Heraeus Deutschland GmbH & Co. KG, Heraeusstr. 12-14, 63450 Hanau, Germany; bernhard.bauer-siebenlist@heraeus.com; 3 National Institute for Research and Development of Optoelectronics Bucharest, Research Institute for Analytical Instrumentation, Donath 67, 400293 Cluj-Napoca, Romania; marin.senila@icia.ro; 4 Innovation Fluides Supercritiques (IFS), Bâtiment INEED, 1 Rue Marc, Seguin, BP16109, 26300 Alixan, France; k.seaudeau@supercriticalfluid.org; 5 Fraunhofer Institute for Chemical Technology (ICT), Joseph-von-Fraunhofer-Str. 7, 76327 Pfinztal, Germany

**Keywords:** extraction, supercritical CO_2_, palladium recycling, polymers, sustainable chemistry, catalysts

## Abstract

Precious metals, in particular Pd, have a wide range of applications in industry. Due to their scarcity, precious metals have to be recycled, preferably with green and energy-saving recycling processes. In this article, palladium extraction from an aluminosilicate-supported catalyst, containing about 2 wt% (weight%) of Pd (100% PdO), with supercritical CO_2_ (scCO_2_) assisted by complexing polymers is described. Two polymers, p(FDA)SH homopolymer and p(FDA-*co*-DPPS) copolymer (FDA: 1,1,2,2-tetrahydroperfluorodecyl acrylate; DPPS: 4-(diphenylphosphino)styrene), were tested with regards to their ability to extract palladium. Both polymers showed relatively low extraction conversions of approximately 18% and 30%, respectively. However, the addition of piperidine as activator for p(FDA-*co*-DPPS) allowed for an increase in the extraction conversion of up to 60%.

## 1. Introduction

Platinum group metals, especially Pd, are extensively used in applications for catalysis, not only in petrochemistry, but also in the fields of automotive and fine chemical synthesis [1,2,3]. The annual demand is constantly increasing, with the demand for Pd up from 242 t in 2010, to over 305 t in 2018 [4]. The scarcity of these metals poses a risk for European countries, which only have very limited primary platinum group resources [1,5,6].

Thus, recycling of precious metals is of high interest. The state-of-the-art recycling processes are either pyro-metallurgical or hydro-metallurgical treatments. The pyro-metallurgical treatment consumes a large amount of energy, due to the high temperatures used, while the hydro-metallurgical treatment has the disadvantage of high wastewater streams, due to the large amounts of leaching solvents [7,8,9]. Accordingly, new alternative recycling methods for precious metals are needed, which are greener, produce smaller amounts of waste, and operate at lower temperatures (<<1450 °C) [9].

Most research focuses on the optimization of the leaching process. For instance, Ding et al. leached Pd from spent catalysts with a hydrochloric acid leaching agent, containing NaCl and FeCl_3_ [10]. By using optimized process conditions (leaching mixture composition, 80 °C and 90 min), a leaching efficiency of 99.5% was reached. Fontana et al. reported a leaching process with aqua regia, followed by the separation of Pd from the leaching solution by solvent extraction, and metal precipitation using NaBH_4_, achieving 83% Pd recovery [11]. Furthermore, Sarioglan et al. leached Pd completely from spent carbon-supported Pd catalysts with an acid leaching solution consisting of HCl and H_2_O_2_ [12].

In contrast, Liu et al. presented a method for recycling Pd from waste printed circuit boards (PCB) without a leaching procedure [13]. Through an initial supercritical water oxidation (SCWO) (425 °C; ≥22.1 MPa) and a post-treatment of the SCWO residue with diluted HCl, extraction-disturbing base metals such as Cu, as well as organic compounds, could be removed. Using this procedure, the Pd concentration in the samples was enriched many times. Afterwards, the Pd was extracted by supercritical CO_2_ (scCO_2_) with acetone as co-solvent, and organic ligand combined with an I_2_-KI combination acting as Pd oxidizer and complexing agent. An extraction of 93.7% of the Pd was achieved.

These recycling methods work, with good Pd recovery rates, but a significant quantity of polluting and environmentally dangerous wastes is still produced [10,11,12,13], or high temperatures are required (425 °C) [13]. Hence, further investigation for a green Pd recycling process is necessary.

Supercritical CO_2_ is a highly available solvent with a tunable solvent power, depending on the applied temperature and pressure. It has a gas-like viscosity and high diffusivity. It is non-toxic, non-flammable, and can be easily separated and recycled after extraction. With a supercritical point that can be achieved under mild conditions (T_C_ = 31 °C, p_C_ = 7.38 MPa) [14,15,16], scCO_2_ is a suitable and attractive solvent for the extraction of Pd under mild operating conditions, and with low waste.

Recently, our group published an article regarding the polymer-assisted extraction of Pd in scCO_2_ from an aluminosilicate-supported catalyst (catalyst Cat D, 2 wt% Pd in the form of PdO (100%)), as well as from pretreated versions of this catalyst [17]. In one case, the catalyst was reduced by H_2_ before extraction (the reduction led to a catalyst composition of Pd^0^ (79%), with a minor amount of PdO (21%)); in the other case, the catalyst was reduced by H_2_, and afterwards oxidized with Cl_2_ (the oxidation led to a catalyst composition of Na_2_PdCl_4_ (85%), with a minor amount of PdO (15%)). The complexing polymers used were p(4VP-*grad*-FDA) and p(4VP-*grad*-FDA)SH (4VP: 4-vinyl pyridine, FDA: 1,1,2,2-tetrahydroperfluorodecyl acrylate). While the extraction conversion of Pd (the amount of Pd removed from the catalyst) without pretreatment was low (≤20%), it was very promising in the case of the oxidized form of the catalyst, reaching up to 73%. Nevertheless, as oxidizing with chlorine generates harmful waste streams, and is difficult to scale-up, a high conversion method for the extraction of Pd from a non-pretreated catalyst would be more advantageous.

The extraction of Pd from a non-pretreated, alumina-supported catalyst containing 61% Pd^0^ and 39% Pd(II) was previously studied by Li et al. [18], using scCO_2_-soluble polymers containing 4-(diphenylphosphino)styrene (DPPS) complexing units. Up to 49% of Pd was extracted from the support, but no details were provided regarding the selectivity of the extraction towards the two oxidation states of Pd (Pd^0^ and Pd(II)), nor regarding the nature of the Pd(II) species.

This study aims to improve the extraction conversion of Pd(II), in the form of PdO, from a non-pretreated catalyst, in order to show that pretreatment of the catalyst (with chlorine) is not required to achieve high extraction conversions through the use of different combinations of polymers and additives, as well as a range of extraction conditions. In this study, the extraction of Pd from a non-pretreated aluminosilicate-supported catalyst (catalyst Cat D, 2 wt% Pd in the form of 100% PdO) in scCO_2_, assisted by complexing polymers and additives is described. For the sake of simplicity, a pristine catalyst (Cat D) was used to avoid complications possibly encountered in the case of a spent catalyst (such as the presence of carbon residues). The extraction process was carried out at mild reaction conditions (40 or 60 °C, 25 or 27 MPa) and had a low waste stream, as most of the components (CO_2_, polymer, Pd, catalyst support) can be potentially recycled later on. Herein, the potential of the scCO_2_ extraction procedure and the influence of the extraction parameters on the extraction conversion and yield are investigated. Promising extraction conversions and yields would show that, combined with previous results [17,18], the scCO_2_ polymer-assisted extraction process can be applied to a wide range of materials.

## 2. Results and Discussion

In this article, extraction screening experiments were performed in a small extraction cell (35 mL) to get a first impression of the extraction ability of the polymer/additive/Pd-catalyst system. Then, in a larger extractor (250 mL), promising extractions were investigated in more detail. In the extraction experiments, the extraction conversion, X_extraction_, refers to the amount of Pd extracted from the catalyst support, and extraction yield, Y_extraction_, refers to the amount of extracted Pd recovered. The amount of Pd extracted used to calculate X_extraction_ and Y_extraction_ was determined by inductively coupled plasma optical emission spectrometry (ICP-OES) in the different sample fractions, as detailed in the materials and methods section and in the Supporting Information. The Pd-balance is the overall mass balance of palladium in the extraction system. The calculations for the Pd-balance are detailed in the materials and methods section.

### 2.1. Design of scCO_2_-Soluble Polymers Capable of Complexing with Pd

**Figure 1:**
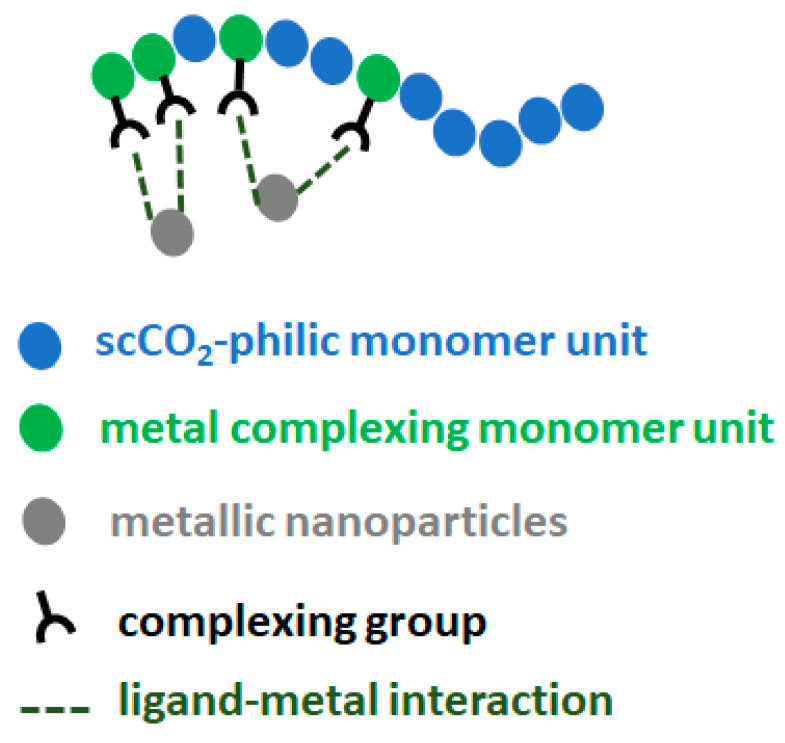
General design of scCO_2_-soluble polymers capable of complexing with Pd.

In this study, fluorinated polymers containing FDA monomer units were chosen as assisting complexing polymers for their good solubility in scCO_2_, while being aware of the harmful potential of this kind of fluorinated polymer. However, as the polymers are to be recycled later on (as will be taken into account in the life cycle analysis, LCA), there should be no exposure of the polymer to the environment. The first polymer was a homopolymer of FDA with a thiol complexing group, p(FDA)SH. The second polymer was a copolymer of FDA with DPPS, providing triphenylphosphine ligands for complexing with Pd (p(FDA-*co*-DPPS)). The polymerization was carried out by a reversible addition-fragmentation chain transfer (RAFT) technique, using a chain transfer agent (CTA) (cf. SI-Chapter 1). The polymers serve as binding agents to bind to the Pd on the catalyst and carry the Pd through the scCO_2_ medium, as the solubility of Pd in scCO_2_ alone is negligible or null. Thus, polymers consisting of one type of scCO_2_-soluble group (FDA) and one or more types of group capable of complexing with Pd (DPPS, -SH) were employed. This general design is shown in Figure 1, with scCO_2_-soluble groups in blue, and the Pd complexing groups in green.

**Figure 2:**
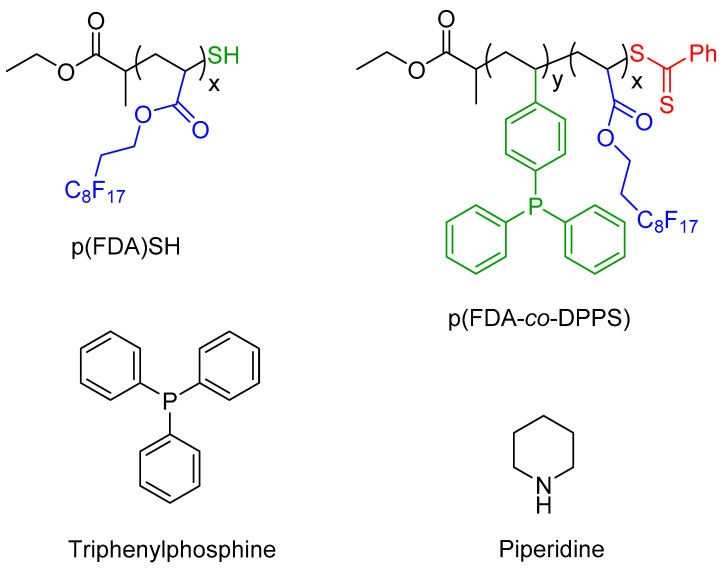
Structures of the complexing polymers and additives used in this study: p(FDA)SH (**top left**), p(FDA-*co*-DPPS) (**top right**), triphenylphosphine (**bottom left**), piperidine (**bottom right**) (scCO_2_-soluble groups are highlighted in blue, complexing groups in green, and the protected thiol group in red).

The two polymers used in this study are presented in Figure 2, with scCO_2_-soluble FDA groups in blue, metal complexing groups in green (thiol group for p(FDA)SH and triphenylphosphine groups for p(FDA-*co*-DPPS)), and protected thiol groups in red. The p(FDA-*co*-DPPS) copolymer bears two different metal complexing groups, given that the protected thiol group is activated by deprotection with an amine (aminolysis). This step was done in situ during the extraction using piperidine (Figure 2), giving two groups capable of complexing with Pd (DPPS units and −SH end group).

### 2.2. Pd Extraction from Catalyst Cat D with Only scCO_2_

**Figure 3:**
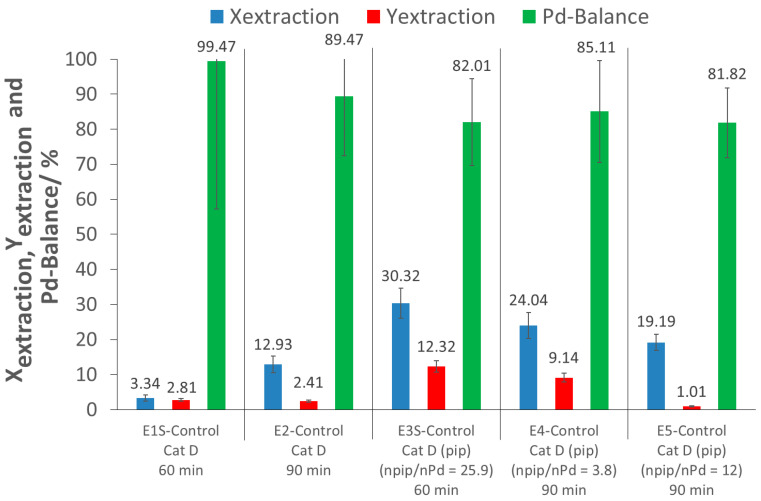
Results of the control tests ((pip): piperidine; npip/nPd: molar ratio of piperidine to Pd; ExS-Control: Screening experiments; Ex-Control: Detailed investigation experiments).

Before using the polymers in extraction, the extraction ability of scCO_2_ alone was investigated. Control tests were performed in both experimental setups (60 min for screening experiments and 90 min for detailed investigations), with and without piperidine (pip), to determine the amount of precious metal extracted using only scCO_2_. The extraction parameters are shown in Table 1, and the extraction results in Figure 3. Further information can be obtained from the supporting information (cf. SI-Chapter 4.1).

For the screening experiment, the extraction of Pd from the pristine catalyst after 60 min (E1S-Control) using only scCO_2_ resulted in a low extraction conversion (where extraction conversion, X_extraction_, is the amount of Pd extracted from the catalyst support) and extraction yield (where extraction yield, Y_extraction_, is the amount of extracted Pd recovered), of 3% each. For the detailed investigation with a 90 min extraction time (E2-Control), an extraction conversion of about 13% was achieved with a low extraction yield of 2%. These low extraction conversions were expected, as there was no complexing agent for the precious metal. The extraction yield of E2-Control being lower than the extraction conversion was due to a loss of Pd during the Pd recovery process. As the Pd-balance was incomplete, some Pd was possibly lost to the atmosphere during CO_2_ depressurization and system flushing after extraction.

Piperidine was used as an in situ activation agent for the deprotection of the terminal dithioester group of the polymers to give a terminal –SH group. Since the –NH group of piperidine can potentially complex with Pd [19], additional control experiments with scCO_2_ and piperidine were performed at different piperidine/Pd ratios (E3S-Control, E4-Control, E5-Control) and extraction times. For the screening experiment, results showed that with 60 min of extraction time, the addition of piperidine, at a molar piperidine/Pd ratio of 25.9, increased the extraction conversion by up to 30% (E3S-Control). In the larger extractor for the detailed investigations, the addition of piperidine at a molar piperidine/Pd ratio of 3.8 and an extraction time of 90 min (E4-control) resulted in an extraction conversion of 24%, while with a piperidine/Pd ratio of 12 (E5-Control), a slightly lower extraction conversion of 19% was achieved. Thus, piperidine has a promoting effect, which is not fully understood at this point, and would deserve further attention. Nevertheless, the extraction conversions (<30%) were still too low for a scale-up and industrial application. Moreover, the extraction yields were even lower (less than 12%), due to loss of Pd during recovery.

### 2.3. Pd Extraction from Catalyst Cat D with PPh_3_

**Figure 4:**
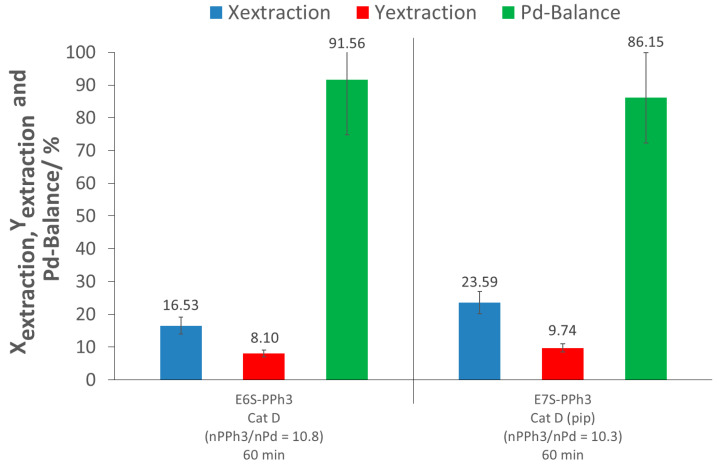
Results of the PPh_3_ extractions ((pip): piperidine; nPPh_3_/nPd: molar ratio of PPh_3_ to Pd; ExS-PPh_3_: Screening experiments).

The previous control experiments showed that for a good extraction of Pd in scCO_2_, an extracting agent (polymer), containing complexing groups, is necessary to bind to the metal. Taking into account the structure of p(FDA-*co*-DPPS), it can be easily identified that the DPPS units mimic the triphenylphosphine ligand (PPh_3_), which is extensively used to form Pd complexes [19,20]. Based on this assumption, the efficiency of the triphenylphosphine molecule as an extracting agent in supercritical CO_2_ was tested (cf. Table 2, Figure 4 and SI-Chapter 4.2). The extraction experiment E6S-PPh_3_ was performed in the presence of PPh_3_ alone for 60 min, without the use of other complexing or activating agents. This test showed the low efficiency of the low molecular weight additive, PPh_3_, as an extracting agent, since the extraction conversion only reached 17%. In addition, the extraction yield was low (8%), due to the same reason as for the control tests (loss of Pd during depressurization and flushing). This result was likely due to the low solubility of PPh_3_ in scCO_2_ [21], which would further decrease if Pd complexation occurred. Despite the low conversion, it was an improvement compared to the control test E1S-Control (in scCO_2_ alone, 60 min). This indicates that the presence of the complexing agent in scCO_2_ increases the extraction of Pd from the aluminosilicate support, simultaneously confirming the complexing ability of the PPh_3_ group for Pd species.

As the p(FDA-*co*-DPPS) extraction experiments involve the in situ activation of the thiol group by piperidine, the addition of both piperidine and PPh_3_ in the scCO_2_ extraction was tested to see if there were any synergic extraction effects between the two complexing groups (E7S-PPh_3_). The results showed a small increase in the extraction conversion, of up to 24% (7% higher than E6S-PPh_3_), while the extraction yield remained nearly constant (10% instead of 8%). However, the amount of extracted palladium and recovered palladium using this combination (E7S-PPh_3_) was actually slightly lower than that measured when piperidine was used alone (E3S-Control) (X: 6% lower; Y: 2% lower), meaning no synergy, and even possibly an unfavorable interaction between the two systems on a molecular level.

### 2.4. Pd Extraction from Catalyst Cat D with Polymer p(FDA)SH

**Figure 5:**
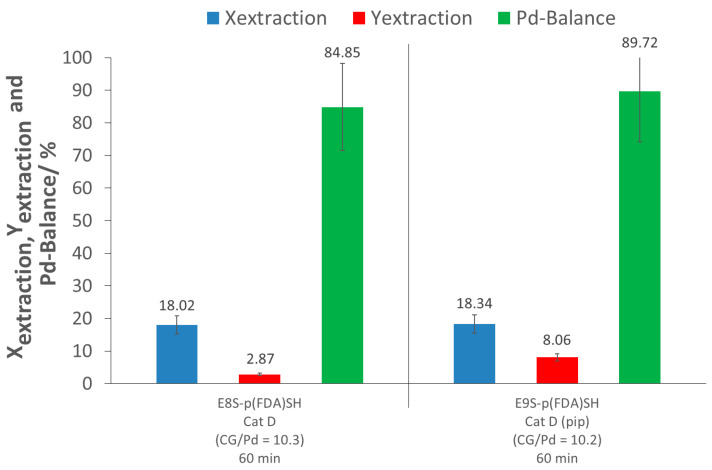
Results of p(FDA)SH extractions ((pip): piperidine; CG/Pd: Complexing group/Pd ratio; ExS-p(FDA)SH: Screening experiments).

After the initial screening of the effect of only scCO_2_, and that of molecular additives in Pd extraction, polymer-assisted extraction was investigated. The first polymer used for this investigation was p(FDA)SH (cf. Figure 2; Table 3; Figure 5; SI-Chapter 4.3). This fluorinated homopolymer is highly soluble in scCO_2_, and contains a thiol end-group, which is a well-known ligand for transition metals, and was expected to be able to complex with Pd(II) species [19,22]. The thiol functionality (-SH) was activated by ex situ aminolysis of the protected polymer in order to have at least one potential complexing group, as the fluorinated units are not able to interact with Pd. The conditions used for the aminolysis of the polymer (N_2_ atmosphere in the presence of PPh_3_ as reducing agent) allowed limiting the disulfide formation during synthesis [23,24]. The thiol group of the resulting p(FDA)SH could potentially couple to give disulfide bonds, but as the polymer is solubilized in scCO_2_ in a predominantly CO_2_ environment (very little oxygen is present) during extraction, the formation of disulfide bonds is unlikely [25]. During storage of the polymer, disulfide formation is possible due to exposure to oxygen [26], but should be limited due to the semi-crystalline nature of the p(FDA) [27], which drastically reduces the mobility of the polymer chains in the solid state [28]. This polymer was used in a molar ratio of 10.3/1 with respect to the PdO present on the catalyst, meaning that a large, ten-fold excess of complexing units was used to perform the metal extraction. The polymer excess of 10.3 was chosen to ensure that a low extraction conversion would not be due to an insufficient number of complexing groups, but to a low polymer complexing ability.

The extraction assisted by the thiol-terminated, fluorinated polymer (E8S-p(FDA)SH) showed limited extraction ability, achieving only 18% extraction conversion. The low efficiency can be explained by a strong interaction of the PdO with the support, as well as by the limited activity of organic ligands towards PdO. The extraction yield was also very low (3%), due to an important loss of Pd (Pd-balance of 85%). A second test was performed using the same polymer, but with the addition of piperidine to verify if this secondary amine is able to improve the solubility of the precious metal in the presence of the fluorinated polymer. The extraction experiment with the combination of p(FDA)SH and piperidine (E9S-p(FDA)SH) did not show any improvement in the extraction conversion (18%), showing an absence of synergic effect between p(FDA)SH and piperidine.

### 2.5. Pd Extraction from Catalyst Cat D with Polymer p(FDA-co-DPPS)

**Figure 6:**
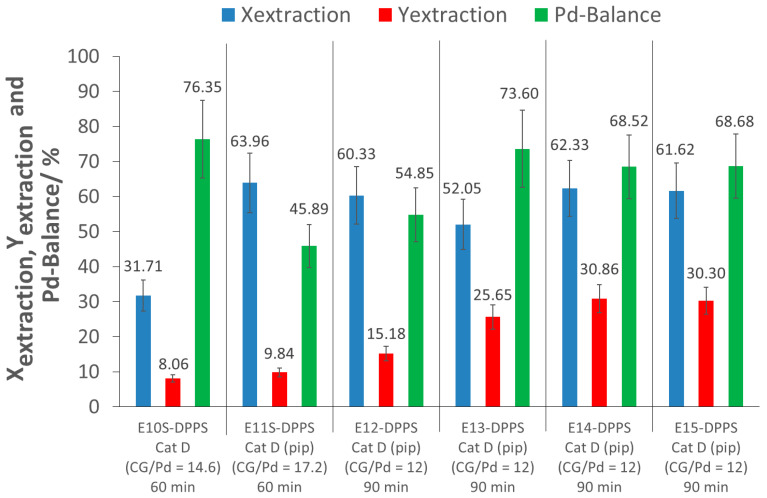
Results of the extractions of Pd with p(FDA-*co*-DPPS) ((pip): piperidine; CG/Pd: Complexing group/Pd ratio; ExS-DPPS: Screening experiments; Ex-DPPS: Detailed investigation experiments).

Extraction tests on the aluminosilicate-supported catalyst Cat D were performed with the polymer p(FDA-*co*-DPPS) (cf. Figure 2). Triphenylphosphine derivatives are well-known for their ability to form complexes with transition metals, especially with Pd [19,20], and DPPS was therefore targeted for use in the extraction experiments. All tests were performed at 25 MPa and 40 °C, with and without activation of the protected thiol group, with 60 min and 90 min of extraction time. The activation was done in situ by a simple addition of piperidine into the extractor, in excess relative to the polymer chains (2.5 to 5-fold molar excess of piperidine relative to the polymer chains depending on the extraction conditions). The extraction parameters and results are shown in Table 4 and Figure 6. Further information can be obtained from the supporting information (cf. SI-Chapter 4.4).

An initial screening extraction experiment with a 60 min extraction time was performed with p(FDA-*co*-DPPS), with a complexing group (CG)/Pd ratio of 14 (large excess of complexing groups) (E10S-DPPS). This extraction gave an interesting result, achieving an extraction of 32% of the precious metal from the support, although the extraction yield was again low (8%), due to Pd loss during depressurization (Pd-balance = 76%). The next step was to try to improve the extraction conversion by activating the protected thiol group of the polymer (the dithiobenzoate end group) via aminolysis, as the thiol group is a well-known ligand for Pd complexation [19,22]. Although deprotection can be performed using several methods, piperidine, a secondary amine, was selected as the aminolyzing agent. This activation process was performed directly in situ in scCO_2_, meaning that piperidine was added directly to the extractor at the beginning of the extraction experiments. This procedure is favored in order to avoid possible disulfide formation (oxidation of thiols). Furthermore, in situ activation was preferred to ex situ aminolysis in order to avoid a synthetic step, and thus optimizing the method for eventual scale-up applications. With the addition of piperidine, while keeping the other extraction parameters constant, the extraction process showed a remarkable improvement, allowing for the removal of 64% of the Pd from the support (E11S-DPPS). This is an increase of over 30% of extracted Pd, when compared to the same experiment without piperidine addition (E10S-DPPS). Nevertheless, the extraction yield was still low (10%) (E11S-DPPS). This promising extraction system was further investigated in more detail in the larger extractor. Four extractions were carried out (E12-DPPS–E15-DPPS) with a 90 min extraction time and a CG/Pd ratio of 12 (large excess of complexing groups), to see if the positive result of E11S-DPPS could also be confirmed in the larger extractor. Extraction conversions between 52 and 62% were achieved, confirming the good Pd complexing ability of p(FDA-*co*-DPPS) in combination with piperidine. This indicates that a positive interaction between the polymer, piperidine, and the catalyst exists, which merits closer investigation. This interaction however, was not present when p(FDA)SH was used, which suggests an interaction specific to the DPPS groups. It is important to note that under the experimental conditions, disulfide formation by oxidative coupling of the thiol group is unlikely. Indeed, the DPPS groups may act as a reducing agent to limit disulfide formation, as triphenylphosphine is used for the reductive cleavage of disulfide bonds [23,24]. It has also been shown that polymer disulfide coupling decreases with increasing polymer molecular weight [28]. Additionally, as the extractions were carried out in CO_2_ atmosphere, there should not be enough oxygen present in the system to favor disulfide formation. However, the in situ formation of thiolactone cannot be completely discarded [25,28,29].

In experiments E12-DPPS–E15-DPPS, two different batches of p(FDA-*co*-DPPS) polymers were investigated, differing in the number of FDA and DPPS monomer units per molecule. Reproducibility of the Pd extractions was confirmed across different polymer batches at a constant complexing group/Pd ratio.

The extraction yields of experiments E11S-DPPS and E12-DPPS–E15-DPPS were low (10 to 31%), as a large amount of Pd was lost during the depressurization and flushing of the cell (Pd-balance in the range of 46 to 74%). Nevertheless, in E13-DPPS to E15-DPPS, approximately 50% of the extracted Pd was recovered. To increase the Pd recovery (extraction yield), a cascade of separators could be used to ensure a slow stepwise depressurization of the CO_2_-Pd-polymer mixture to atmospheric pressure, minimizing this loss of Pd. During depressurization, the transition of CO_2_ from the supercritical domain to gas state occurs, separating the Pd-polymer mixture from CO_2_, as the polymer is insoluble in gaseous CO_2_.

**Figure 7:**
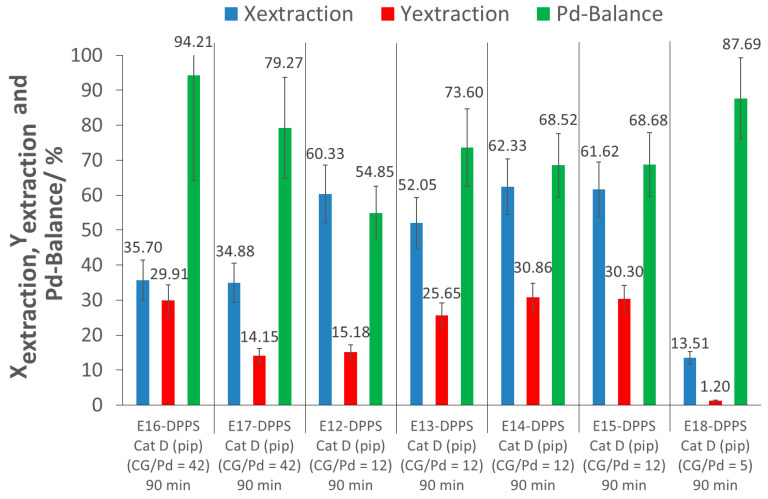
Results of the extractions of Pd with p(FDA-*co*-DPPS) at decreasing CG/Pd ratios ((pip): piperidine; CG/Pd: Complexing group/Pd ratio; Ex-DPPS: Detailed investigation experiments).

**Figure 8:**
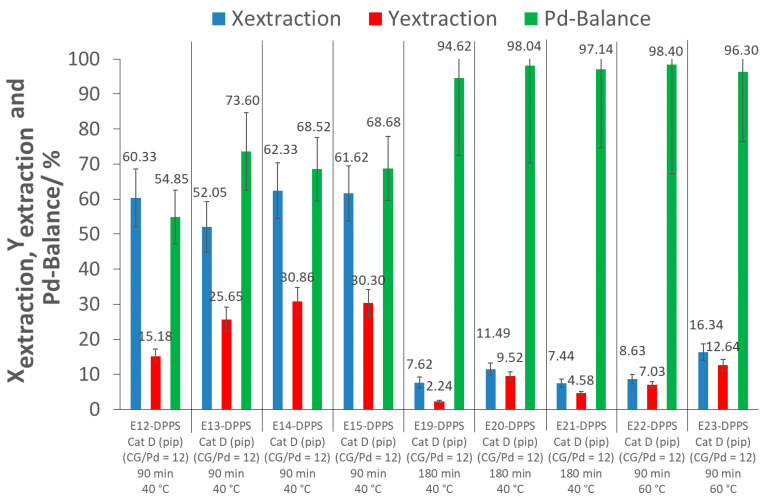
Results of the extractions of Pd with p(FDA-*co*-DPPS) at different extraction times and temperatures ((pip): piperidine; CG/Pd: Complexing group/Pd ratio; Ex-DPPS: Detailed investigation experiments).

To have a better understanding of the influence of the CG/Pd ratio, as well as the influence of the extraction time, extraction experiments with p(FDA-*co*-DPPS) were performed at much higher (42) and lower (5) CG/Pd ratios, and at a much longer extraction time of 180 min. Extraction tests were also performed at 60 °C instead of 40 °C. The extraction parameters and results are shown in Table 5, Figure 7 and Figure 8 (further information in SI-Chapter 4.4).

As can be observed in experiments E16-DPPS and E17-DPPS, a large increase of the CG/Pd ratio did not improve the extraction, but rather led to a poorer extraction performance as extraction conversions of only 35% were obtained, much lower than in the case of CG/Pd = 12 (approximately 60%). This might have been due to intermolecular polymer interaction as a result of the higher polymer concentration, making it more difficult for the polymers to interact with the catalyst, due to more competition between the polymers, and thus inhibiting complexation.

A decrease of the CG/Pd ratio to 5 (E18-DPPS) resulted in a lower extraction conversion as well, likely due to an insufficient amount of polymers for the extraction of the Pd nanoparticles.

The extraction yields obtained across extractions performed at different CG/Pd ratios were inconsistent, and varied between 1 and 31% (E12-DPPS to E18-DPPS). As indicated previously, a loss of Pd during the recovery process contributes to this variability. From the set of experiments E12-DPPS to E18-DPPS, a CG/Pd ratio of about 12 appeared to provide a good excess of complexing groups, yielding good extraction results.

As can be seen from Figure 8, an increase in the extraction temperature to 60 °C resulted in a decrease in the extraction conversions and yields, regardless of the pressure used, 25 MPa (E22-DPPS) or, to increase the solubility of p(FDA-*co*-DPPS), at 27 MPa (E23-DPPS). Further tests will have to be performed at other temperatures to verify this effect and explain this behavior.

When the extractions were extended to 180 min (doubled extraction time) at 40 °C, a decrease in extraction conversion and extraction yield was observed as well (E19-DPPS to E21-DPPS). The cause of the lower extraction efficiency with a longer extraction time cannot be explained at this point, and will require a more detailed investigation into the effects of the operating conditions and the extraction mechanism. From our present results, at an extraction temperature of 40 °C, it appears that an extraction time of 90 min is optimal for the larger extractor.

It can also be observed from Figure 8 that the Pd-balances were high in the cases where extraction conversions were low. As mentioned previously, the loss of Pd during the depressurization step and flushing of the extractor led to low extraction yields. The fact that the Pd-balances were high when extraction conversions were low supports this explanation. If the Pd was not extracted from the catalyst, it remained on the support. However, Pd that was extracted by the polymer was able to flow through the sCO_2_ medium, and could leave the extraction system during depressurization.

From this preliminary parameter study, it appears that the initially chosen conditions of 40 °C and 90 min of extraction time in the larger extractor gave the best results for the Pd extraction from an aluminosilicate-supported catalyst using p(FDA-*co*-DPPS) as complexing polymer, at a CG/Pd ratio of 12, with piperidine as in situ activator. An increase in the extraction temperature from 40 °C to 60 °C, a large extension of the extraction time from 90 to 180 min, or a significant increase or decrease of the CG/Pd ratio did not improve extraction conversion. Of course, a more complete parameter study using a wider range of operating conditions will be required to fully analyze the individual and combined effects of extraction time, temperature, and the CG/Pd ratio on extraction efficiencies.

## 3. Materials and Methods

### 3.1. Reactants

#### 3.1.1. Complexing Polymers

The synthesis and characterization of the complexing polymers, p(FDA)SH and p(FDA-*co*-DPPS), are described in the Supporting Information (SI-Chapter 1) [30,31,32].

#### 3.1.2. Catalyst

The Catalyst D (Cat D) used for the extraction experiments was provided by Heraeus. It is a pristine Pd/aluminosilicate (2.083 wt% Pd; 100% PdO) catalyst. The detailed characterization of this catalyst is provided in the Supporting Information (SI-Chapter 2) [17,33].

### 3.2. Extraction Procedures

Extraction experiments were performed in two different extractors, differing mostly in their size (V_small extraction cell_ = 35 mL; V_larger extractor_ = 250 mL). In the small extraction cell, the extraction experiments with 60 min of extraction time were performed as a quick screening of the polymers’ Pd complexing ability at standard operating conditions (T = 40 °C; p = 25 MPa). Longer extraction experiments, as well as experiments under other experimental conditions, were performed in the larger extractor (detailed investigations).

#### 3.2.1. Chemicals

The piperidine used for thiol group activation (aminolysis) was either received from Sigma Aldrich (purity of 99%) for the screening experiments, or from Merck (purity of ≥99%) for the detailed investigations. Triphenylphosphine (PPh_3_) was received from Sigma Aldrich with a purity of 99%. CO_2_ bottles were provided either by Air Liquide (CO_2_ SFE 5.2 grade at 99.9% purity) for the screening experiments or from Linde (2.5 grade at purity 99.5%) for the detailed investigations. All chemicals were used as received.

#### 3.2.2. Extraction Procedure for Screening Experiments

The catalyst, polymer, and if applicable piperidine, were placed in a 35 mL stainless steel extraction cell (Top Industrie, France), which was then tightly closed. The extraction cell was equipped with magnetic stirring, a PTFE-coated magnetic stir bar, a rupture disk, a pressure transducer, and two stainless steel filters (PORAL, class 7) positioned at the inlet and outlet of the set-up. The extraction cell was heated with a mantle monitored by a proportional-integral-derivative temperature controller with a thermocouple (type K) inside the extraction medium. An ISCO model no. 260D automatic syringe pump (with an internal pressure transducer), thermostated by a water/isopropanol mixture delivered by a LAUDA RE206 circulating pump, was used to pressurize the extraction cell with CO_2_.

The ISCO pump was stabilized at 27 MPa and 35 °C. Afterwards, the extraction cell was filled with CO_2_ until 25 MPa was reached while heating at 40 °C (≈39 mL CO_2_ delivered by the ISCO pump). The extraction was performed under magnetic stirring at 100 rpm for one hour at 25 MPa and 40 °C (batch conditions). After the one hour extraction time was completed, the cell was flushed with ≈ 160 mL CO_2_ delivered by the ISCO pump (26 MPa and 35 °C in the ISCO pump) at a flow rate of about 0.6–1.2 mL/min, and the exiting CO_2_/polymer/Pd mixture was bubbled into water contained in a plastic flask at the outlet of the extraction set-up.

**Figure 9:**
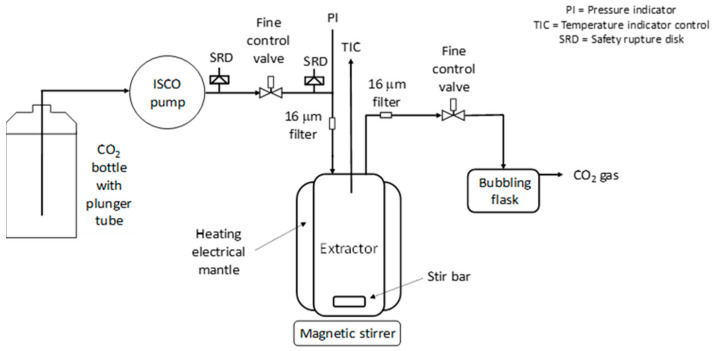
Scheme of screening experiment apparatus.

Then, the extraction cell was opened and the catalyst was recovered (Sample: ExS-B; where “E” stands for “Experiment”, “x” for the “experiment number”, “S” for ”screening experiment”, while “B” is the sample assignment to its origin in the experimental setup). The cell was cleaned with acetone, which was then collected and evaporated (Sample: ExS-C). The bubbling water sample, containing the precipitated polymer (potentially loaded with Pd) was taken as Sample ExS-A. Furthermore, the tubes, valves, and filters were cleaned with acetone, which was also collected and evaporated afterwards (Sample: ExS-D). The samples were analyzed by ICP-OES (cf. SI-Chapters 3.1 and 3.2), to determine the amount of Pd extracted from the catalyst support (Conversion (X)), and recovered (Yield (Y)). A scheme of the screening extraction apparatus is shown in Figure 9. Table 6 shows the samples taken after extraction.

#### 3.2.3. Extraction Procedure for Detailed Investigations

The catalyst, polymer, and if applicable piperidine, were placed in the extractor (250 mL stainless steel reactor fitted with a 10 µm sinter) in the desired amounts. The extractor was closed and the magnetic agitation was set to 250 rpm. The desired temperature (40 or 60 °C) was adjusted via a heating cartridge. Afterwards, an HPLC pump (Type: Wadose from Wagner) connected with a Coriolis flow meter (Type: Bronkhorst; M13-AGD-33-O-S; 6–600 g/h CO_2_) was used to feed the CO_2_ into the extractor (5 g/min) until the desired pressure was obtained: 25 MPa (at 40 °C ≈ 217 g CO_2_; at 60 °C ≈ 193 g CO_2_) or 27 MPa (at 60 °C ≈ 202 g CO_2_). The extractor outlet was closed by a stop valve (exit valve). After the desired pressure was reached, the CO_2_ feed was stopped, and the inlet to the extractor was closed using another stop valve (inlet valve). Afterwards, the extraction was performed under magnetic stirring at 250 rpm for 90 or 180 min, at 25 or 27 MPa, and 40 or 60 °C (batch extraction).

After the desired extraction time was completed, the exit valve of the extractor was opened slowly, so that polymer, Pd, and CO_2_ could flow into the separator (stainless steel vessel with sapphire windows), and bubble into an acetone bath. The vent and input of the separator were controlled using two fine valves, one at the extractor and one at the separator outlet. The HPLC pump fed CO_2_ to the extractor at about 5 g/min to keep the extractor at 25 or 27 MPa, and the temperature was kept at 40 or 60 °C. Approximately 250 to 1500 g of CO_2_ was fed through the extractor as a flushing medium to ensure that all the polymer was transported from the extractor to the separator. As the transported polymer could be observed as a continuous polymer precipitation in the acetone bath in the separator (viewed through the sapphire windows), this gave a visual indication of when the flushing process was complete. This is the reason for the variation in the quantity of CO_2_ used during flushing in the different extraction tests. In the separator, a pressure of 5 MPa and room temperature (20 °C) were maintained so that CO_2_ in the gas state could leave through a reverse osmosis membrane (Filmtec SW 30, thin film polyamide membrane (PA + PS), Separation limit: NaCl: 99.6%) (Sample: Exe; where “E” stands for “experiment”; “x” for the “experiment number”; and “e” for the sample assignment to its origin in the experimental setup). The Pd and the polymer were collected in the separator acetone bath. After flushing, the pressure in the extraction apparatus was released slowly and the heating was switched off.

The supported catalyst was then recovered from the extractor (Sample: Exc), as well as the acetone sample from the separator (Sample: Exa). Afterwards, the separator, including tubing to the extractor, (Sample: Exb) and the extractor itself (Sample: Exd) were washed with acetone separately.

**Figure 10:**
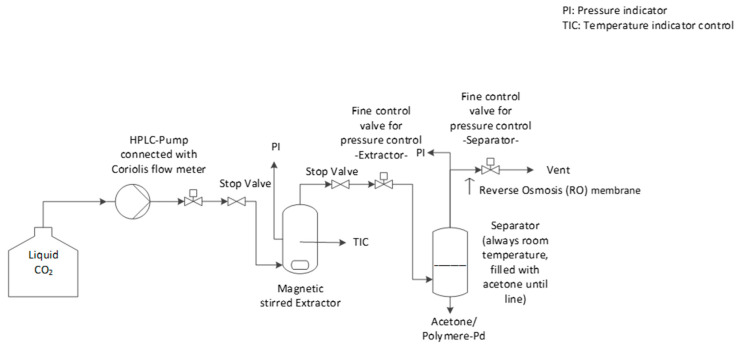
Scheme of the extraction apparatus for detailed investigations.

For all acetone-containing samples, the acetone was evaporated under a fume hood. Afterwards, the samples were analyzed by ICP-OES (cf. SI-Chapters 3.1 and 3.2) to determine the amount of Pd extracted from the catalyst support (Conversion (X)) and recovered (Yield (Y)). A scheme of the detailed investigation test apparatus is shown in Figure 10. Table 7 describes the samples taken after extraction.

#### 3.2.4. Calculation of the Desired Reactant Quantities

To calculate the correct ratio of catalyst, polymer, piperidine, and CO_2_, initially an excess of complexing groups to Pd of 10–17 for the screening experiments (exact adjustment was impossible due to the small batches) and of 12 for the detailed investigations were used. Later on, this ratio was varied to much larger (42) and lower (5) values.

This initial excess of complexing groups compared to the Pd content was an estimated value, and was used to ensure that the initial results were a consequence of the polymer complexing abilities, and not due to an insufficient quantity of complexing groups.

The amounts of complexing groups in the different polymers were determined via ^1^H-NMR (cf. SI-chapter 1.2), as well as the polymer molecular weight.

The p(FDA)SH homopolymer (M_n_ = 5735 g/mol) contained only one complexing group per polymer, the -SH group.

The p(FDA-*co*-DPPS) copolymers contained several monomer units of DPPS complexing groups, plus, in the case of thiol group activation, one -SH complexing group. The p(FDA-*co*-DPPS) copolymers used in this work were synthesized in different batches, and therefore contained different amounts of DPPS groups and different molecular weights. The first batch contained seven DPPS groups per polymer chain (M_n_ = 11,600 g/mol), while the second one contained 10 DPPS groups per polymer chain (M_n_ = 16,723 g/mol).

With this knowledge, the polymer/Pd molar ratio was calculated by dividing the complexing group/Pd ratio by the amount of complexing groups in one polymer chain (cf. Equation (1)).

(1) $$\frac{n_{polymer}}{n_{Pd}}=\frac{\mathrm{Complexing}\;\mathrm{group}/\mathrm{Pd}\;ratio}{Amount\;of\;complexing\;groups\;per\;polymer\;chain}$$

where n_polymer_ is the moles of polymer to be used in the extraction test, and n_Pd_ is the moles of Pd on the pristine catalyst.

To determine the appropriate polymer concentration in scCO_2_ (c_pol_(%)) to use, where the polymer is completely soluble under the extraction conditions, cloud point (CP) curves of a 1 wt% mixture of the three polymers in scCO_2_ were measured (cf. SI-Chapter 1.3, calculation with Equation (2)). Good solubility of all three polymers at the extraction conditions (40 and 60 °C and 25 or 27 MPa) was found for a concentration of 1 wt% polymer in scCO_2_. Thus, for the detailed investigations, polymer concentrations of less than 1 wt% were chosen (cf. reactant ratio tables in SI). For the screening experiments, higher polymer concentrations were used (cf. reactant ratio tables in SI) in accordance with previous results [18], where at 25 MPa and 40 °C, both p(FDA)SH and p(FDA-*co*-DPPS) were completely soluble in scCO_2_, at least up to 10 wt% of polymer in CO_2_.

(2) $$c_{pol}\left(\%\right)=\frac{m_{pol}}{m_{CO2}}\times 100$$

where *m_pol_* is the mass of polymer used in the extraction test, and m_CO_2__ is the mass of CO_2_ fed to the extractor.

The amounts of polymer and catalyst required for each extraction were calculated taking into account the polymer concentration in scCO_2_, the amount of CO_2_ needed to reach 25 or 27 MPa, the polymer/Pd molar ratio, and the catalyst Pd loading.

Piperidine, used as thiol group activator (in situ aminolysis of the dithiobenzoate end group of p(FDA-*co*-DPPS)), was initially used in a molar excess of 2.5, and subsequently used in a molar excess of 5, with respect to the polymer.

### 3.3. Calculation of Conversion (X), Yield (Y), and Pd-Balance

Conversion (X) is defined as the amount of Pd removed from the catalyst. The yield (Y) is defined as the amount of Pd recovered after extraction, not including the Pd remaining on the catalyst.

During the study, it was found that large quantities of Pd extracted from the support remained in the extractors after extraction, and were not transferred during flushing into the water bath/separator. As this Pd was no longer fixed to the support (the catalyst Cat D could be easily and completely removed from the extractor after extraction) and could be easily recovered by washing the extractors with acetone, this Pd was counted as extracted in this study. The calculations of the extraction conversion and the extraction yield are shown in Equations (3) and (4), respectively.

(3) $$X_{extraction}=\frac{m_{Pd,Cat}-m_{Pd,Cat\;after\;extraction}}{m_{Pd,Cat}}\times 100\%$$

(4) $$Y_{extraction}=\frac{(m_{Pd,\mathrm{water}\;\mathrm{bath}/separator}+m_{Pd,\;\mathrm{extractor}}+m_{Pd,RO-membrane})}{m_{Pd,Cat}}\times 100\%$$

The Pd-Balance was calculated as the quotient of the amount of Pd found in any part of the extraction apparatus or on the catalyst after extraction, and the amount of Pd on the catalyst before the extraction (cf. Equation (5)).

(5) $$Pd-Balance=\frac{(m_{Pd,\mathrm{water}\;\mathrm{bath}/separator}+m_{Pd,\;\mathrm{extractor}}+m_{Pd,RO-membrane}+m_{Pd,Cat\;after\;extraction\;})}{m_{Pd,Cat\;}}\times 100\%\;$$

where *m_Pd,Cat_* is the mass of palladium on the pristine catalyst, *m_Pd,Cat after extraction_* is the mass of palladium on the catalyst after extraction (Sample: ExS-B or Exc), *m*_*Pd*,extractor_ is the mass of palladium recovered from the extractor (Sample: ExS-C or Exd), *m*_*Pd*,water bath/*separator*_ is the mass of Pd recovered from the water bath/separator (mass of Pd recovered from bubbling in water/acetone + mass of Pd recovered from separator and tubings acetone wash) (Sample: ExS-A + ExS-D or Exa + Exb), and *m_Pd,RO-membrane_* is the mass of palladium recovered from the reverse osmosis membrane in the separator outlet (Exe).

## 4. Conclusions

In this study, an effective process for the polymer-assisted supercritical CO_2_ extraction of Pd(II) from a non-pretreated aluminosilicate-supported catalyst consisting of 100% PdO is presented. The p(FDA-*co*-DPPS) copolymer, activated in situ with piperidine, led to good extraction conversions of approximately 60% at 40 °C and 25 MPa. These results show that a pretreatment of the catalyst Cat D is not required to achieve promising extraction conversions, with extraction results from a non-optimized process already comparable to those achieved with a pretreatment of the catalyst [17]. Through the use of the right combination of complexing polymers and piperidine, as well as the fine-tuning of extraction conditions, the harmful waste streams generated during pretreatment of the catalyst with chlorine can be eliminated.

In screening experiments using p(FDA-*co*-DPPS) without piperidine or p(FDA)SH, much lower extraction conversions were obtained. Likewise, the use of scCO_2_ alone or scCO_2_ with piperidine and/or triphenylphosphine molecular ligands resulted in only low extraction rates or none at all, confirming the necessity of using a macromolecular CO_2_-soluble complexing agent that is able to bind to the palladium and transport it through the supercritical media.

A preliminary extraction parameter study was performed, showing that an increase in the temperature (from 40 to 60 °C), a doubling of the extraction time (from 90 min to 180 min), or a strong increase (from 12 to 42) or decrease (from 12 to 5) of the complexing group/Pd ratio did not result in higher Pd extraction conversions.

The presented method showed good reproducibility in terms of extraction conversions, even across different polymer batches. However, as only half of the extracted Pd could be recovered, with the remaining Pd likely lost to the atmosphere during the recovery process, there is room for improvement.

A more comprehensive parameter study will be necessary to optimize and improve the extraction conversion and extraction yield obtained using this p(FDA-*co*-DPPS)/piperidine system. For the optimization of the extraction process, a deeper investigation of the extraction mechanism (synergy effect) in the case of the p(FDA-*co*-DPPS)/piperidine system is needed. The separation of Pd from the polymer, and therefore the recycling of the polymer, is not part of this study. Several methods, e.g., electrochemical deposition, are currently under investigation, and will be presented in further works. Furthermore, the life cycle analysis (LCA) of this new Pd extraction procedure is in progress.

## Figures and Tables

**Table 1:** Extraction parameters for control tests (ExS-Control: Screening experiments; Ex-Control: Detailed investigation experiments).

Experiment Number	Polymer	Activation Reagent	Piperidine/Pd Molar Ratio	p/MPa	T/°C	Extraction Time/min	m_CO_2_,flushing_/g
E1S-Control	-	-	-	25	40	60	145
E2-Control	-	-	-	25	40	90	1500
E3S-Control	-	Piperidine	25.9	25	40	60	145
E4-Control	-	Piperidine	3.8	25	40	90	1500
E5-Control	-	Piperidine	12	25	40	90	600

**Table 2:** Extraction parameters for PPh_3_ extraction tests (ExS-PPh_3_: Screening experiments).

Experiment Number	Polymer	Activation Reagent	PPh_3_/Pd Molar Ratio	p/MPa	T/°C	Extraction Time/min	m_CO_2_,flushing_/g
E6S-PPh_3_	-	PPh_3_	10.8	25	40	60	145
E7S-PPh_3_	-	PPh_3_/Piperidine	10.3	25	40	60	145

**Table 3:** Extraction parameters for extractions performed with polymer p(FDA)SH (ExS-p(FDA)SH: Screening experiments).

Experiment Number	Polymer	Activation Reagent	Complexing Group (CG)/Pd Molar Ratio	p/MPa	T/°C	Extraction Time/min	m_CO_2_,flushing_/g
E8S-p(FDA)SH	p(FDA)_11_SH	-	10.3	25	40	60	145
E9S-p(FDA)SH	p(FDA)_11_SH	Piperidine	10.2	25	40	60	145

**Table 4:** Extraction parameters for extractions performed with polymer p(FDA-*co*-DPPS) (ExS-DPPS: Screening experiments; Ex-DPPS: Detailed investigation experiments).

Experiment Number	Polymer	Activation Reagent	Complexing Group (CG)/Pd Molar Ratio	p/MPa	T/°C	Extraction Time/min	m_CO_2_,flushing_/g
E10S-DPPS	p(FDA_18_-*co*-DPPS_7_)	-	14.6	25	40	60	145
E11S-DPPS	p(FDA_18_-*co*-DPPS_7_)	Piperidine	17.2	25	40	60	145
E12-DPPS	p(FDA_18_-*co*-DPPS_7_)	Piperidine	12	25	40	90	1500
E13-DPPS	p(FDA_18_-*co*-DPPS_7_)	Piperidine	12	25	40	90	-
E14-DPPS	p(FDA_26_-*co*-DPPS_10_)	Piperidine	12	25	40	90	250
E15-DPPS	p(FDA_26_-*co*-DPPS_10_)	Piperidine	12	25	40	90	400

**Table 5:** Extraction parameters for extractions performed with polymer p(FDA-*co*-DPPS) at different CG/Pd ratios, extraction times and temperatures (Ex-DPPS: Detailed investigation experiments).

Experiment Number	Polymer	Activation Reagent	Complexing Group (CG)/Pd Molar Ratio	p/MPa	T/°C	Extraction Time/min	m_CO_2_,flushing_/g
E12-DPPS	p(FDA_18_-*co*-DPPS_7_)	Piperidine	12	25	40	90	1500
E13-DPPS	p(FDA_18_-*co*-DPPS_7_)	Piperidine	12	25	40	90	-
E14-DPPS	p(FDA_26_-*co*-DPPS_10_)	Piperidine	12	25	40	90	250
E15-DPPS	p(FDA_26_-*co*-DPPS_10_)	Piperidine	12	25	40	90	400
E16-DPPS	p(FDA_26_-*co*-DPPS_10_)	Piperidine	42	25	40	90	250
E17-DPPS	p(FDA_26_-*co*-DPPS_10_)	Piperidine	42	25	40	90	600
E18-DPPS	p(FDA_26_-*co*-DPPS_10_)	Piperidine	5	25	40	90	600
E19-DPPS	p(FDA_18_-*co*-DPPS_7_)	Piperidine	12	25	40	180	1500
E20-DPPS	p(FDA_26_-*co*-DPPS_10_)	Piperidine	12	25	40	180	400
E21-DPPS	p(FDA_26_-*co*-DPPS_10_)	Piperidine	12	25	40	180	400
E22-DPPS	p(FDA_26_-*co*-DPPS_10_)	Piperidine	12	25	60	90	600
E23-DPPS	p(FDA_26_-*co*-DPPS_10_)	Piperidine	12	27	60	90	400

**Table 6:** Sample nomenclature for the screening experiments.

Sample Name	Sample Origin
ExS-A	Bubbling water solution
ExS-B	Catalyst after extraction
ExS-C	Washing of extractor with acetone
ExS-D	Washing tubes, valves, and filters with acetone

(E: Experiment; x: Experiment number; S: Screening experiment; “A–D”: sample assignment corresponding to sample origin in the experimental setup).

**Table 7:** Sample nomenclature for the detailed investigations.

Sample Name	Sample Origin
Exa	Acetone bath separator
Exb	Washing of separator and pipes with acetone
Exc	Catalyst after extraction
Exd	Washing of extractor with acetone
Exe	Reverse osmosis (RO) membrane

(E: Experiment; x: Experiment number; “a–e”: sample assignment to the sample origin in the experimental setup).

## Data Availability

The data presented in this study are available in the Supporting Information.

## Supplementary Materials

Supplementary information is available online. Figure S1. ^1^H-NMR of p(FDA)_11_SH after precipitation. Figure S2. ^1^H-NMR of p(FDA_18_-*co*-DPPS_7_) post-precipitation. Figure S3. ^1^H-NMR of p(FDA_26_-*co*-DPPS_10_) post-precipitation. Figure S4. Cloud point (CP) curve of the polymer p(FDA_11_)SH at 1 wt% in CO_2_. Figure S5. Cloud point (CP) curve of the polymer p(FDA_18_-*co*-DPPS_7_) at 1 wt% in CO_2_. Figure S6. Cloud point (CP) curve of the polymer p(FDA_26_-*co*-DPPS_10_) at 1 wt% in CO_2_. Figure S7. SEM-EDX image of Cat D. Figure S8. EDX and element compositions at the surface of Cat D. Figure S9. EDX and element compositions inside Cat D (fractured bead). Figure S10. XPS spectrum Pd 3d of Cat D. Figure S11. TEM and particle size distribution of Cat D. Figure S12. Nitrogen adsorption-desorption isotherms and pore size distribution of Cat D. Table S1. Elemental composition determined by XPS (atomic percentages). Table S2. Specific surface area and average pore diameter determined by BET. Table S3. Digestion program for samples. Table S4. Reactant ratios used for the control experiments (cat: catalyst; pip: piperidine; CG: Complexing group; ExS-Control: Screening experiments; Ex-Control: Detailed investigation experiments). Table S5. Pd content of the samples from the control experiments (ExS-A to D: Samples from screening experiments; Exa to e: Samples from detailed investigation experiments). Table S6. Percentage of Pd in samples of control experiments (ExS-Control: Screening experiments; Ex-Control: Detailed investigation experiments; ExS-A to D: Samples from screening experiments; Exa to e: Samples from detailed investigation experiments). Table S7. Extraction results and errors of control experiments (ExS-Control: Screening experiments; Ex-Control: Detailed investigation experiments). Table S8. Reactant ratios used for PPh_3_ extraction experiments (cat: catalyst; pip: piperidine; CG: Complexing group; ExS-PPh_3_: Screening experiments). Table S9. Pd content of the samples from PPh_3_ extraction experiments (ExS-A to D: Samples from screening experiments). Table S10. Percentage of Pd in samples for PPh_3_ extraction experiments (ExS-PPh_3_: Screening experiments; ExS-A to D: Samples from screening experiments; Exa to e: Samples from detailed investigation experiments). Table S11. Extraction results and errors of PPh_3_ extraction experiments (ExS-PPh_3_: Screening experiments). Table S12. Reactant ratios used for extraction experiments with p(FDA)SH (cat: catalyst; pip: piperidine; CG: Complexing group; ExS-p(FDA)SH: Screening experiments). Table S13. Pd content of the samples from extraction experiments with p(FDA)SH (ExS-A to D: Samples from screening experiments). Table S14. Percentage of Pd in the samples for extraction experiments with p(FDA)SH (ExS-p(FDA)SH: Screening experiments; ExS-A to D: Samples from screening experiments; Exa to e: Samples from detailed investigation experiments). Table S15. Extraction results and errors of p(FDA)SH extraction experiments (ExS-p(FDA)SH: Screening experiments). Table S16. Reactant ratios used for extraction experiments with p(FDA-*co*-DPPS) (40 °C, 25 MPa) (cat: catalyst; pip: piperidine; CG: Complexing group; ExS-DPPS: Screening experiments; Ex-DPPS: Detailed investigation experiments). Table S17. Pd content of the samples from the extraction experiments with p(FDA-*co*-DPPS) (40 °C, 25 MPa) (ExS-A to D: Samples from screening experiments; Exa to e: Samples from detailed investigation experiments). Table S18. Percentage of Pd in the samples of the extraction experiments with p(FDA-*co*-DPPS) (40 °C, 25 MPa) (ExS-DPPS: Screening experiments; Ex-DPPS: Detailed investigation experiments; ExS-A to D: Samples from screening experiments; Exa to e: Samples from detailed investigation experiments). Table S19. Extraction results and errors of the extraction experiments with p(FDA-*co*-DPPS) (40 °C, 25 MPa) (ExS-DPPS: Screening experiments; Ex-DPPS: Detailed investigation experiments). Table S20. Reactant ratios used for extraction experiments with p(FDA-*co*-DPPS) at parameter screening (cat: catalyst; pip: piperidine; CG: Complexing group; Ex-DPPS: Detailed investigation experiments). Table S21. Pd content of the samples from the extraction experiments with p(FDA-*co*-DPPS) at parameter screening (Exa to e: Samples from detailed investigation experiments). Table S22. Percentage of Pd in the samples of the extraction experiments with p(FDA-*co*-DPPS) at parameter screening (Ex-DPPS: Detailed investigation experiments; ExS-A to D: Samples from screening experiments; Exa to e: Samples from detailed investigation experiments). Table S23. Extraction results and errors of the extraction experiments with p(FDA-*co*-DPPS) at parameter screening (Ex-DPPS: Detailed investigation experiments).

## Sample Availability

Samples of the compounds are not available from the authors.
