# Supplementary material for: Supercritical CO2 Extraction of Palladium Oxide from an Aluminosilicate-Supported Catalyst Enhanced by a Combination of Complexing Polymers and Piperidine
Source: Molecules. 2021 Jan 28;26(3):684. doi: 10.3390/molecules26030684 (PMC7865370; doi:10.3390/molecules26030684)
Supplement: Supplementary file 1 [file molecules-26-00684-s001.pdf]

Supporting information

Supercritical CO_2 Extraction of Palladium Oxide From an Aluminosilicate-Supported Catalyst Enhanced by a Combination of Complexing Polymers and Piperidine

Andrea Ruiu [1], Bernhard Bauer-Siebenlist [2], Marin Senila [3], W. S. Jennifer Li [1], Karine Seaudeau-Pirouley [4], Patrick Lacroix-Desmazes [1,*] and Thorsten Jänisch [5,*]

[1] ICGM, Univ Montpellier, CNRS, ENSCM, 34095 Montpellier, France; andrea1.ruiu@gmail.com (A.R.); wing-sze.li@enscm.fr (W.S.J.L.);
[2] Heraeus Deutschland GmbH & Co. KG, Heraeusstr. 12-14, 63450 Hanau, Germany; bernhard.bauer-siebenlist@heraeus.com (B.B.-S.)
[3] National Institute for Research and Development of Optoelectronics Bucharest, Research Institute for Analytical Instrumentation, Donath 67, 400293 Cluj-Napoca, Romania; marin.senila@icia.ro (M.S.)
[4] Innovation Fluides Supercritiques (IFS), Bâtiment INEED, 1 Rue Marc, Seguin, BP16109, 26300 Alixan, France; k.seaudeau@supercriticalfluid.org (K.S.-P.)
[5] Fraunhofer Institute for Chemical Technology (ICT), Joseph-von-Fraunhofer-Str. 7, 76327 Pfinztal, Germany
*Correspondence: patrick.lacroix-desmazes@enscm.fr (P.L.-D.); tjaenisch82@gmail.com (T.J.)

1. Complexing Polymers

1.1. Synthesis of the Complexing Polymers

The complexing polymers, p(FDA)SH homopolymer and p(FDA-*co*-DPPS) copolymer, were synthesized via reversible addition-fragmentation chain transfer (RAFT) polymerization.

1.1.1. Chemicals for RAFT-Polymer Synthesis

FDA (1,1,2,2-tetrahydroperFluoroDecyl Acrylate, > 98%, Boc Science), TFT (α, α, α–trifluorotoluene, > 98%, Aldrich), DPPS (4-(DiPhenylPhosphino)Styrene, 97%, Aldrich), pentane (> 99%, VWR), and 1,2-trichlorotrifluoroethane (CFC-113, Freon 113, Aldrich, 99%) were used as received.

AIBN (2,2′-Azobis(2-methylpropionitrile)) from Fluka, purity 98%, was further purified by recrystallization in methanol and dried under vacuum before use.

The chain transfer agent (CTA) (ethyl-2-(phenylcarbonothioylthio)propionate) was synthesized and purified, as previously reported in the literature [1].

1.1.2. Synthesis of p(FDA)SH Homopolymer

FDA (40 g, 0.0771 mol), CTA (2.1407 g, 0.0084 mol), AIBN (0.4146 g, 0.0025 mol) and TFT (42 mL) were added in a Schlenk flask. The mixture was stirred magnetically, and bubbled for 40 min with N_2. Afterwards, the polymerization was initiated by heating the Schlenk flask in an oil bath at 65 °C. After 2 weeks, the reaction was stopped and left to return to room temperature. The polymer was precipitated in 600 mL of pentane three times, and then dried under vacuum overnight. The precipitated polymer was aminolyzed by the addition of piperidine (5 eq.) and PPh_3 (3 eq.) in TFT, with the mixture stirred magnetically and bubbled for 40 min with N_2. The aminolysis reaction proceeded for 3 h. The p(FDA)SH polymer was precipitated in pentane three times and the polymer was

dried under vacuum overnight. After drying, the polymer was recovered as a fine white powder (68% yield).

1.1.3. Synthesis of p(FDA-co-DPPS) Copolymer

FDA (42.5 g, 0.0820 mol), DPPS (7.5 g, 0.0260 mol), CTA (1.305 g, 0.0051 mol), AIBN (0.2525 g, 0.0015 mol), and TFT (54 mL) were added in a Schlenk flask. The mixture was stirred magnetically and bubbled for 40 min with N_2. Afterwards, the polymerization was initiated by heating the Schlenk flask in an oil bath at 65 °C. After 96 h, the reaction was stopped and left to return to room temperature. The polymer was precipitated in 600 mL of pentane three times, and was dried under vacuum overnight. After drying, the polymer was recovered as a fine pink powder (61% yield).

1.2. Polymer Characterization

The polymer composition was determined by ^{1}H-NMR spectroscopy with a Bruker Avance 400 MHz spectrometer at room temperature. The spectrum was recorded by dissolving 10 mg of polymer in 0.5 mL of CFC-113 with C_6D_6 capillary tubes. The experimental conditions for recording ^{1}H-NMR spectrum were as follows: flip angle 30°, acquisition time 4 s, pulse delay 1 s, and 32 scans.

p(FDA$_{11}$)SH (used in E8S-p(FDA)SH and E9S-p(FDA)SH)

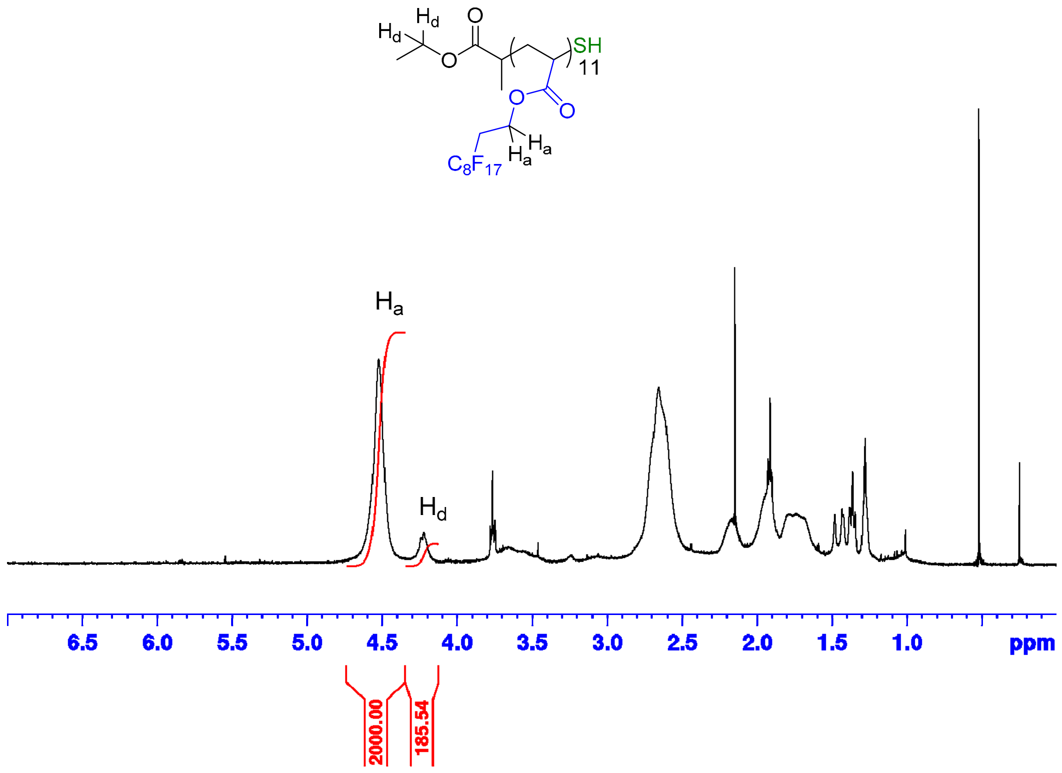

Figure S1. ^{1}H-NMR of p(FDA)$_{11}$SH after precipitation.

The degree of polymerization (DP_{FDA}) of the monomer unit (FDA) was calculated based on the following formula, where H_i corresponds to the integral of the protons i in the ^{1}H-NMR spectrum (cf. Figure S1):

$$DP_{FDA} = \frac{H_a/2}{H_d/2} = 10.81$$

$$M_{n,precipitated\ p(FDA)_{11}SH}\ (g/mol) = DP_{FDA} \times M_{FDA} + M_{Post-aminolysis\ CTA} = 5735\ \ g/mol$$

with M_{FDA} = 518.17 g/mol, $M_{Post\text{-}aminolysis\ CTA}$ = 134.19 g/mol.

p(FDA$_{18}$-co-DPPS$_7$) (used in E10S-DPPS, E11S-DPPS, E12-DPPS, E13-DPPS and E19-DPPS)

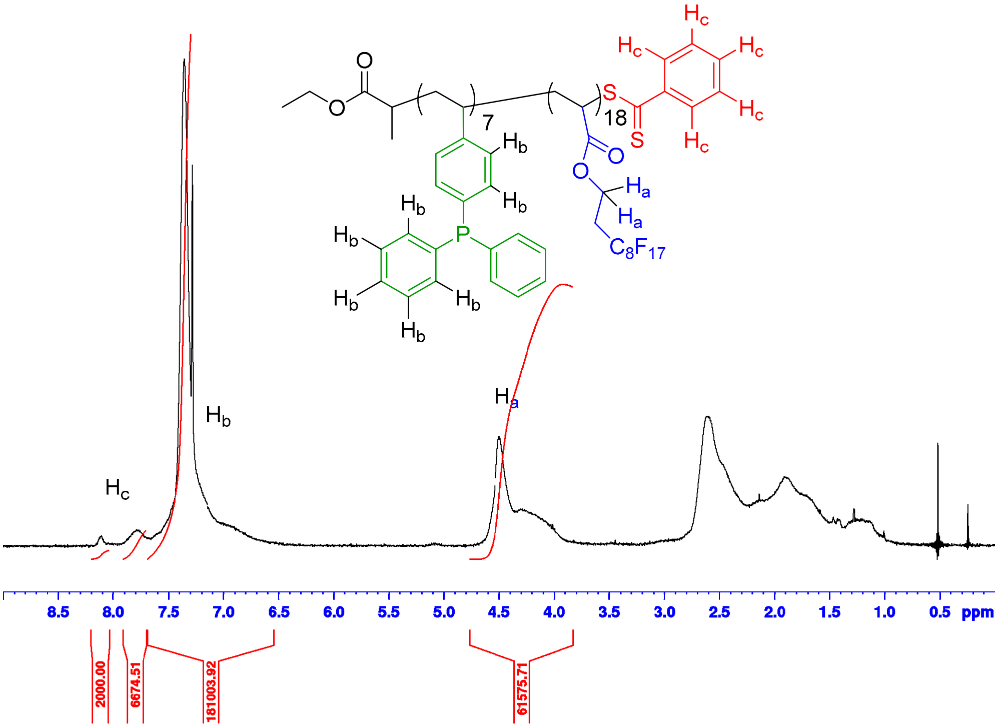

Figure S2. ^{1}H-NMR of p(FDA$_{18}$-*co*-DPPS$_7$) post-precipitation.

The degrees of polymerization of the two different monomer units (DP_{FDA} and DP_{DPPS}) were calculated based on the following formula, where H_i corresponds to the integral of the protons i in the ^{1}H-NMR spectrum (cf. Figure S2):

$$DP_{DPPS} = \frac{H_b/14}{H_c/5} = 7.45$$

$$DP_{FDA} = \frac{H_a/2}{H_c/5} = 17.75$$

$$M_{n,precipitated\ p(FDA_{18}-co-DPPS_7)}\ (g/mol) = DP_{DPPS} \times M_{DPPS} + DP_{FDA} \times M_{FDA} + M_{CTA}$$

$$= 11600\ \ g/mol$$

with M_{FDA} = 518.17 g/mol, M_{DPPS} = 288.32 g/mol, M_{CTA} = 254.36 g/mol.

p(FDA$_{26}$-*co*-DPPS$_{10}$) (used in E14-DPPS–E18-DPPS and E20-DPPS–E23-DPPS)

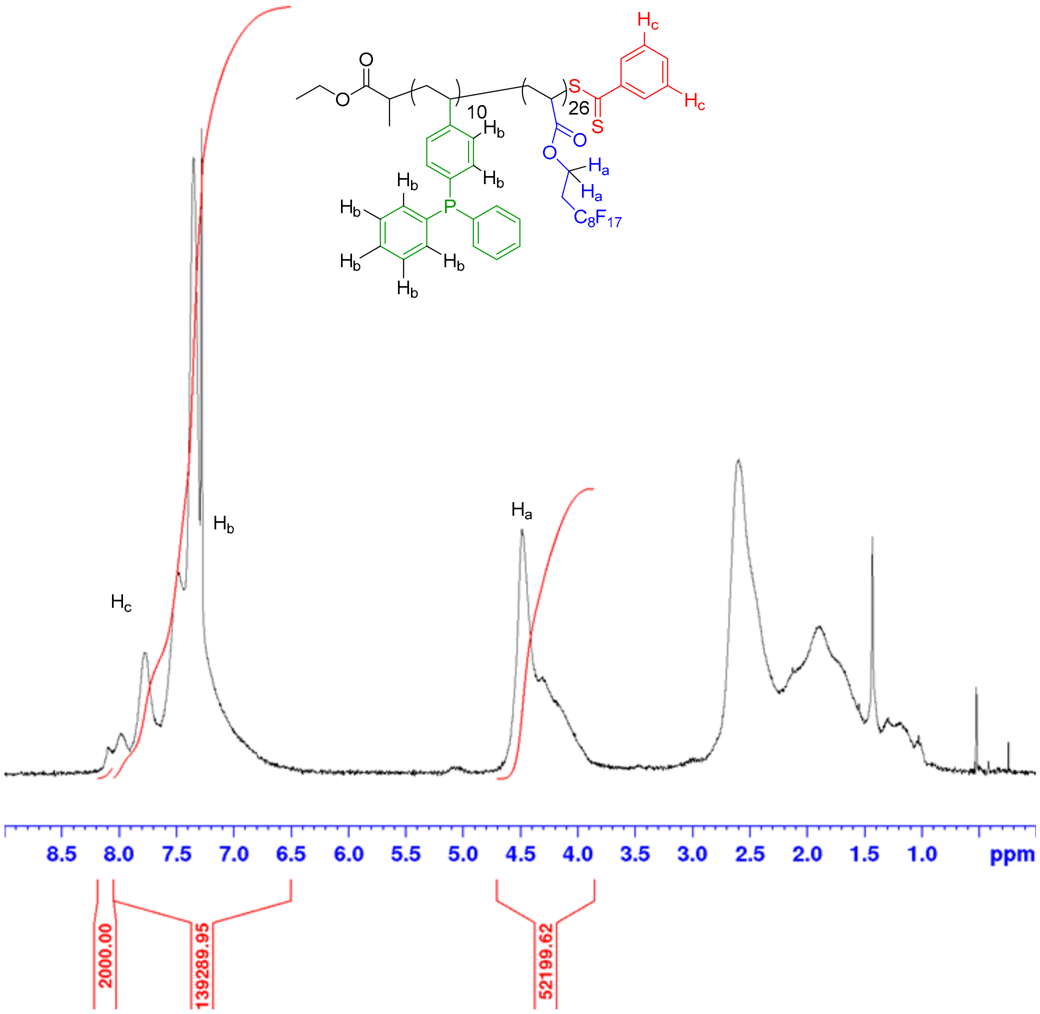

Figure S3. ^{1}H-NMR of p(FDA$_{26}$-*co*-DPPS$_{10}$) post-precipitation.

The degrees of polymerization of the two different monomer units (DP_{FDA} and DP_{DPPS}) were calculated based on the following formula, where H_i corresponds to the integral of the protons i in the ^{1}H-NMR spectrum (cf. Figure S3):

$$DP_{DPPS} = \frac{H_b/_{14}}{H_c/_{2}} = 9.78$$

$$DP_{FDA} = \frac{H_a/_{2}}{H_c/_{2}} = 26.34$$

$$M_{n,precipitated\ p(FDA_{26}-co-DPPS_{10})}\ (g/mol) = DP_{DPPS} \times M_{DPPS} + DP_{FDA} \times\ M_{FDA} +\ M_{CTA}$$

$$= 16723\ \ g/mol$$

with M_{FDA} = 518.17 g/mol, M_{DPPS} = 288.32 g/mol, M_{CTA} = 254.36 g/mol.

1.3. Cloud Point Curves of Polymers in scCO$_2$

The cloud points were measured with a polymer content of 1 wt% in CO_2 with the following procedure.

Cloud-point measurements were carried out in a high pressure, variable volume view cell equipped with a sapphire window on the end for visual observations. The cell was equipped with a pressure transducer and an internal thermocouple. It was thermostated by a water/isopropanol mixture delivered by a Lauda RE206 circulating pump. CO_2 was delivered by an ISCO 260D automatic syringe pump. A total of 50–55 mg of polymer was weighed and transferred to the cell along with a clean magnetic stir bar at a starting cell volume of 6.39 mL. Subsequently, the cell was fed with CO_2 at about 25 °C and 10.9 MPa. Then, the cell was heated to 65 °C (taking care to adjust the volume of the cell in order to stay below a pressure of 35 MPa; safety rupture disk at 50 MPa) and then cooled by steps of 5 °C down to 25 °C. Cloud points (one-phase/two-phase transition) were obtained by decreasing the pressure of the cell by increasing the cell volume through a hand-driven piston after 20 min of stirring at a given temperature. The uncertainty of the cloud point pressure was ±0.5 MPa.

As can be seen in Figures S4 – S6, all three polymers were completely soluble in $scCO_2$ at the extraction conditions, 25 or 27 MPa and 40 or 60 °C.

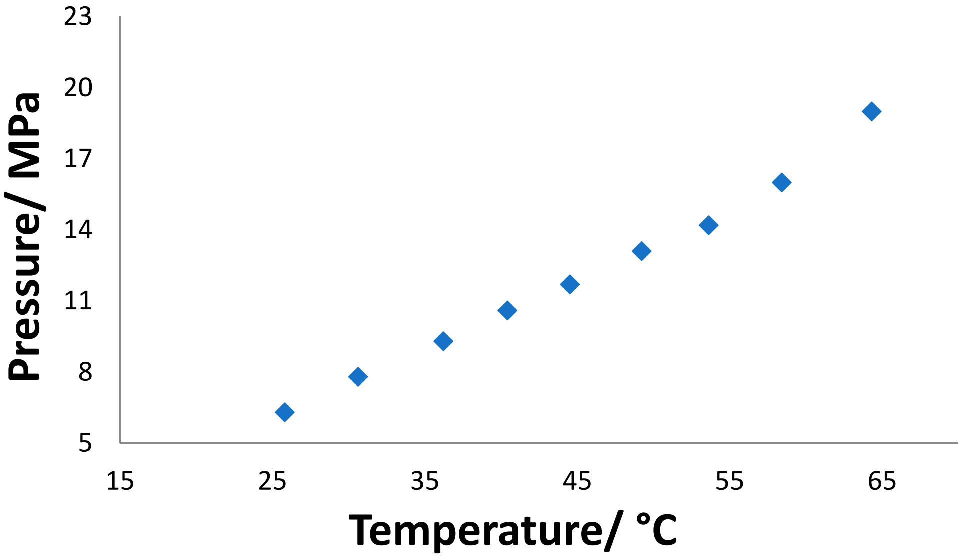

Figure S4. Cloud point (CP) curve of the polymer p(FDA_{11})SH at 1 wt% in CO_2.

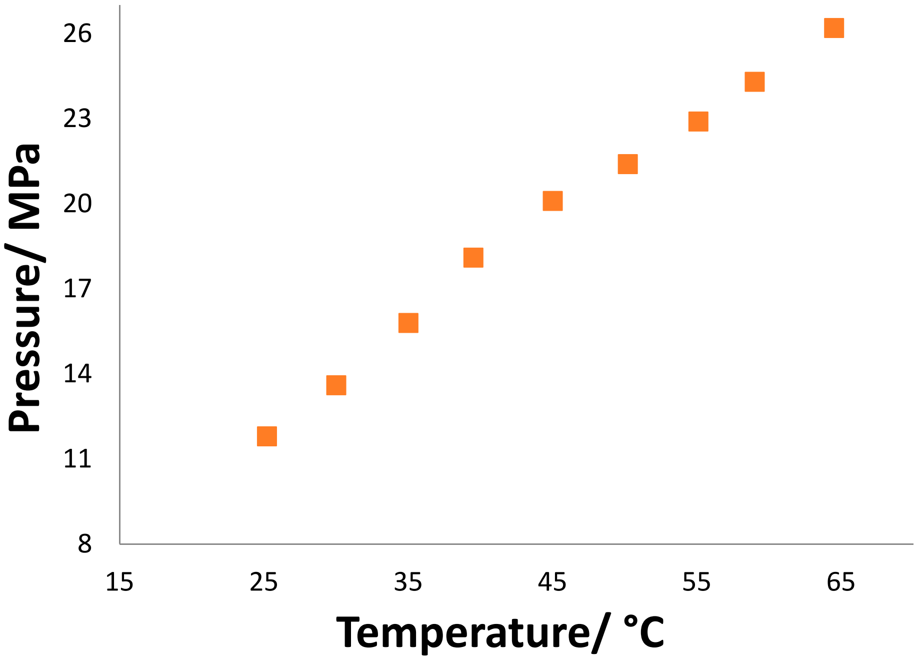

Figure S5. Cloud point (CP) curve of the polymer p(FDA$_{18}$-*co*-DPPS$_7$) at 1 wt% in CO_2.

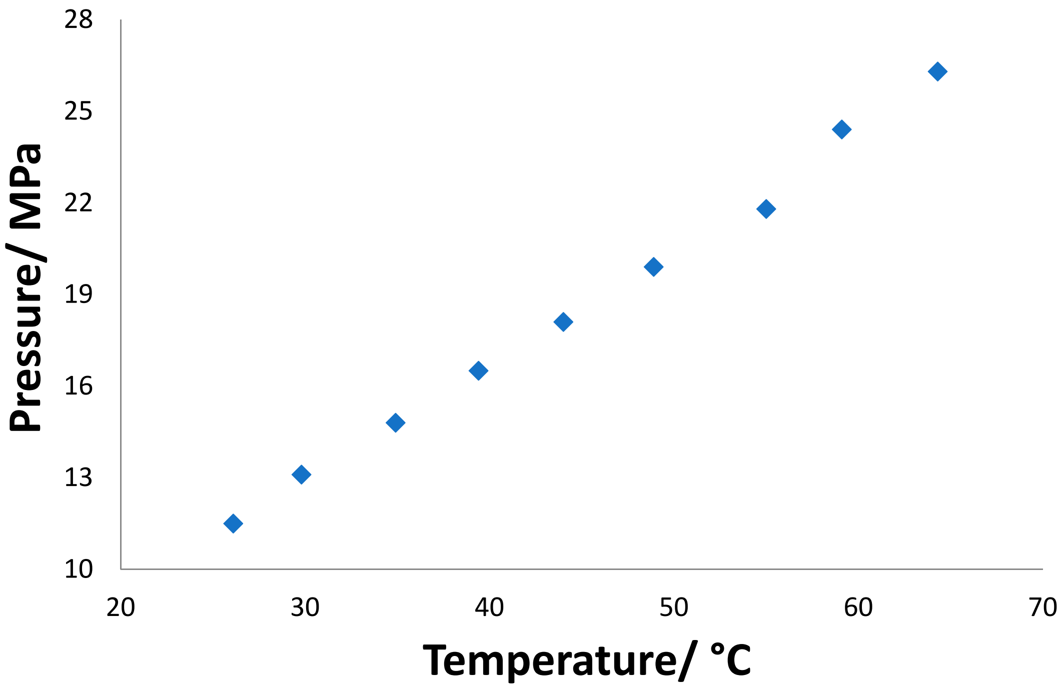

Figure S6. Cloud point (CP) curve of the polymer p(FDA$_{26}$-*co*-DPPS$_{10}$) at 1 wt% in CO_2.

2. Characterization of Catalyst Cat D

The catalyst characterization was reported previously [2], as the same batch of catalyst was used in the present study. The Cat D characterization is shown here again, just for reference.

2.1. SEM-EDX

The SEM-EDX analyses were done with a ZEISS EVO HD15 coupled with an EDX ATzec (Oxford instrument) apparatus. The catalyst Cat D (2 wt% Pd) was deposited as a

powder on a carbon based, electrically conductive, double sided adhesive. The samples were prepared by carbon metallization to perform the analysis. This process increases the C % atomic by about 2%.

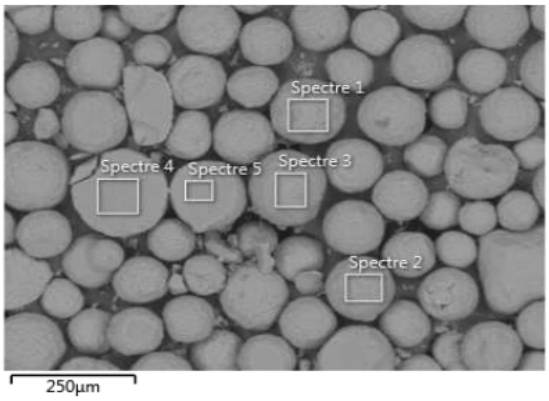

Figure S7. SEM-EDX image of Cat D.

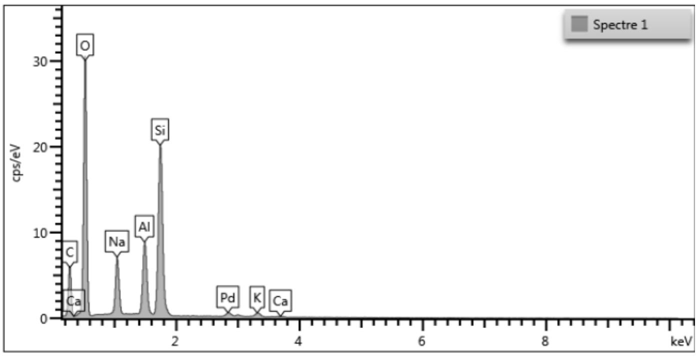

Element	% Mass	% Atomic
C	5.16	8.70
O	42.73	54.35
Na	7.30	6.47
Al	10.33	7.79
Si	28.87	20.92
K	1.77	0.92
Ca	0.36	0.19
Pd	3.47	0.67

Figure S8. EDX and element compositions at the surface of Cat D.

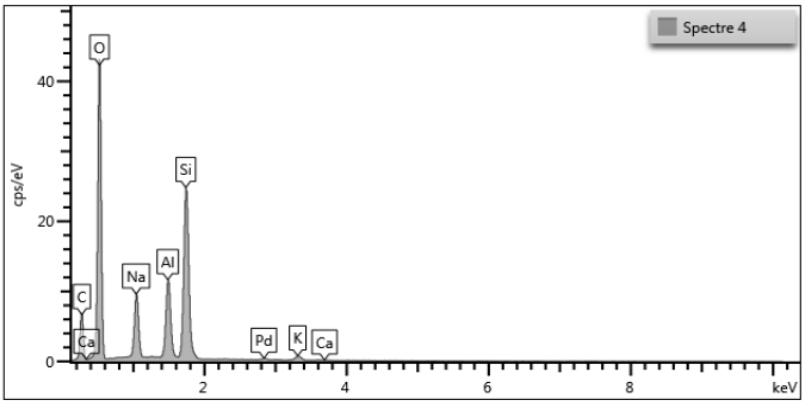

Element	% Mass	% Atomic
C	5.10	8.37
O	46.40	57.14
Na	7.97	6.83
Al	10.32	7.53
Si	27.04	18.97
K	1.56	0.79
Ca	0.26	0.13
Pd	1.37	0.26

Figure S9. EDX and element compositions inside Cat D (fractured bead).

The average size of the catalyst was 80 micrometers, measured by SEM-EDX (cf. Figure S7). From the SEM-EDX studies performed on Cat D, it was found that about 72% of the Pd was present on the surface of the catalyst (cf. Figure S8), but the precious metal was also present in the interior of the support (cf. Figure S9):

$(Pd_{surface}/Al_{surface})/(Pd_{surface}/Al_{surface} + Pd_{inside}/Al_{inside}) = (3.47/10.33)/(3.47/10.33 + 1.37/10.32) = 71.7\%$.

2.2. XPS

XPS measurements were carried out with a THERMO Escalab spectrometer, using focused monochromatic Al K$_\alpha$ radiation (hν = 1486.6 eV). Peaks were recorded with constant pass energy of 20 eV. Charge neutralization was used for all the acquisitions. The pressure in the analysis chamber was around 5 × 10^{-11} MPa. Short acquisition time spectra were recorded before each experiment to check that the samples did not suffer from degradation during the measurements. The binding energy scale was calibrated using the C 1 s peak at 285.0 eV from the hydrocarbon contamination invariably present. The curves fit for core peaks were obtained using a minimum number of components.

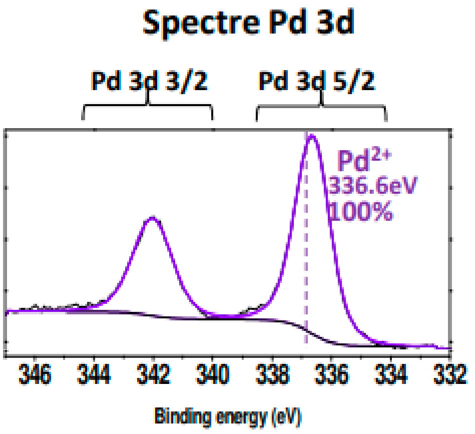

Figure S10. XPS spectrum Pd 3d of Cat D.

Table S1. Elemental composition determined by XPS (atomic percentages).

Catalyst	Pd	Al	Si	O	C	Na	K	Cl
Cat D	0.8	9	24	48	12	5	1	-

The XPS characterization (Figure S10, Table S1) allowed for the study of the oxidation state of the precious metal on the aluminosilicate support. For palladium, the Pd 3d spectrum was recorded. The Pd 3d spectrum corresponds to a doublet, due to the spin orbit splitting of the d orbital. Hence, the Pd has two peaks named Pd 3d 5/2 and Pd 3d 3/2. For Cat D (cf. Figure S10), the presence of a unique peak at 336.6 eV, typical for Pd(II)O species (100%) was observed [3].

2.3. TEM

TEM images were obtained with a Jeol 1200EXII transmission electron microscope at an operating voltage of 100 kV, with images captured with a Quemesa camera from Olympus Soft Imaging Solutions. Supports were crushed into powder form, and embedded into an Embed 812 resin, which was then microtomed using an Ultramicrotome Ultracut UCT from Leica Microsystems, equipped with a DiATOME ultra diamond knife, and placed on a 300-mesh copper grid for TEM analysis.

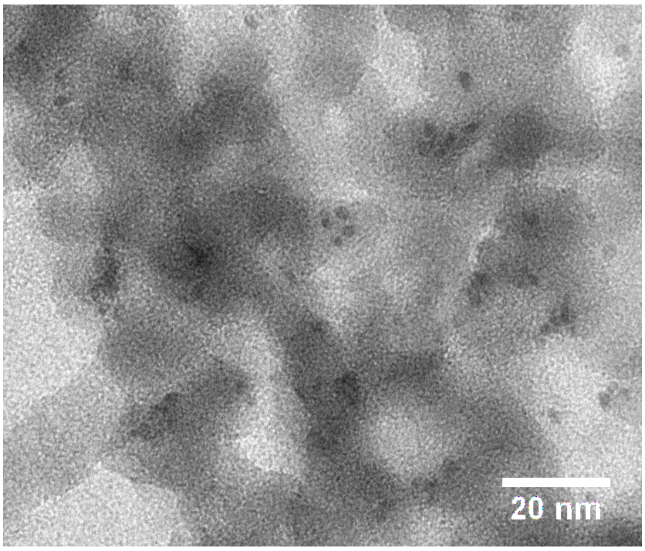

**Number-Average Size (D_n)**	
Average (nm)	2.7
Std (nm)	1.1
Median (nm)	2.3
**Mass-Average Size (D_w)**	
D_w (nm)	4.4
**Polydispersity Index (PDI)**	
D_w/D_n	1.67

Figure S11. TEM and particle size distribution of Cat D.

For Cat D (pristine catalyst, 100% PdO), TEM studies showed nanoparticles with an average diameter of 2.7 nm, and with a relatively low dispersity in size (cf. Figure S11).

2.4. Nitrogen Adsorption–Desorption Isotherms (BET)

The mesopore size distributions and specific surface area were determined by nitrogen adsorption–desorption isotherms (BET) using an ASAP-2020 physisorption analyzer (Micromeritics). The samples were heated at 120 °C under reduced pressure (10^{-3} MPa) for 24 h before the analysis.

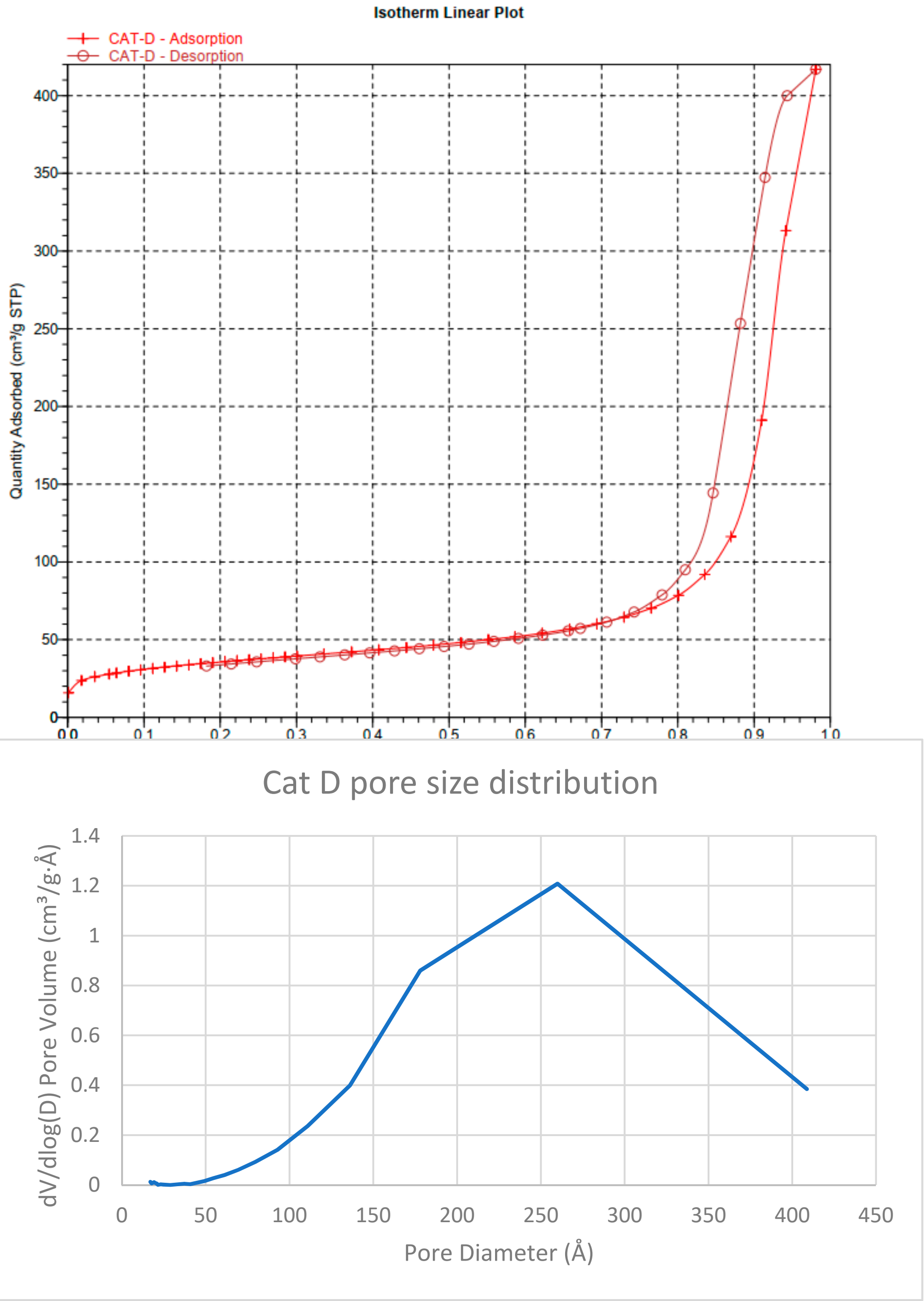

Figure S12. Nitrogen adsorption–desorption isotherms and pore size distribution of Cat D.

Table S2. Specific surface area and average pore diameter determined by BET.

Catalyst	Specific Surface Area	Average Pore Diameter (Adsorption Isotherm)
Cat D	122 m^2/g	21 nm

For catalyst Cat D, a surface area of 122 m^2/g was measured with an average pore diameter of 21 nm (Figure S12, Table S2), large enough to allow the fluorinated polymers (ca. 10 nm diameter micelles [4,5]) to enter the catalyst pores.

3. Materials and Methods

3.1. ICP-OES Analysis for Pd Content Determination in the Samples

The content of Pd in the different samples (pristine catalyst, bubbling water/acetone solution with Pd and polymer, catalyst recovered in the extraction cell after extraction, acetone cleaning solutions of the extraction cell and the separator, acetone cleaning solution of tubes, valve and filters, and reverse osmosis membrane after extraction) collected from the extraction experiments at the two sites was measured by inductively coupled plasma optical emission spectrometry (ICP-OES) after sample digestion using mineral acids (aqua regia mixture).

An inductively coupled plasma optical emission spectrometer (ICP-OES) OPTIMA 5300DV (Perkin Elmer, USA) was used to determine Pd at the wavelengths of 340.458 nm and 324.270 nm. The operating conditions employed for ICP-OES determination were 1300 W RF power, 15 l/min plasma flow, 2.0 l/min auxiliary flow, 0.8 l/min nebulizer flow, and 1.5 mL/min sample uptake rate. Instrument calibration was performed with standard solutions prepared from commercial Pd solutions of 1000 mg/l from Merck, Darmstadt, Germany.

Digestion of solid samples was carried out in PTFE vessels of a microwave digestion oven with temperature control (Speedwave MWS-3 from Berghof GmbH). The temperature program shown in Table S3 was applied for all samples.

Table S3. Digestion program for samples.

Step	1	2	3	4
T /°C	100	200	100	25
Time/min	10	20	5	5

Digestion of sample ExS-A (the bubbling water solution containing polymer and Pd):

Sample ExS-A (up to 140 mL) was transferred to a Berzelius glass and concentrated to approx. 25 mL on a hotplate by evaporation. Then, 24 mL of aqua regia was added to the plastic bottle to leach the Pd remaining in the bottle. It was then added to the concentrated sample on the hotplate. The solution (aqua regia + sample) was boiled for 2 h to reduce to the digested sample volume (cf. SI-Chapter 4). The sample was then cooled to room temperature and filtered on a cellulose filter (circles, diam. 125 mm; Whatman) using glass funnels into a fitting volumetric flask. The final sample was analyzed for Pd by ICP-OES, and the mass concentration of Pd in the sample (c_{Pd} (mg/l)) was obtained.

Digestion of catalyst Cat D, sample ExS-B and Exc (the catalyst recovered after extraction):

100 – 200 mg of sample was weighed in PTFE vessels. Then, 15 mL of aqua regia was added and introduced in the microwave oven. Afterwards, the digestion program from Table S3 was applied. After digestion, the sample was cooled down to room temperature and filtered on a cellulose filter (circles, diam. 125 mm; Whatman) using glass funnels into a fitting volumetric flask. The sample was then diluted to the digested sample volume using ultrapure water (cf. SI-Chapter 4). The resulting solution was analyzed by ICP-OES to obtain the mass concentration of Pd in the sample (c_{Pd} [mg/l]).

Digestion of sample ExS-C, ExS-D, Exa, Exb, and Exd (acetone bubbling/cleaning solutions):

Samples were transferred to PTFE vessels. For this, the bottle sent for analysis containing the sample was washed with aqua regia (14 mL) and introduced into the microwave oven. The digestion program from Table S3 was applied and after digestion, the sample was cooled down to room temperature and filtered on a cellulose filter into a fit-

ting volumetric flask. The sample was diluted to the digested sample volume with ultrapure water (cf. SI-Chapter 4). The mass concentration of Pd in the sample was analyzed by ICP-OES (c_{Pd} [mg/l]).

Digestion of sample Exe (RO-membrane):

The sample (membrane) was introduced to a PTFE vessel in the microwave oven and 14 mL of aqua regia was added. Then, the digestion program shown in Table S3 was applied. The digested sample was filtered, after cooling down to room temperature, into a fitting volumetric flask, and was diluted to the digested sample volume with ultrapure water (cf. SI-Chapter 4). Afterwards the mass concentration of Pd in the sample was analyzed by ICP-OES (c_{Pd} [mg/l]).

3.2. Calculation of Pd Mass in the Samples

The Pd ppm content, ppm_{Pd}, of each sample was calculated using Equation (S1).

$$ppm_{Pd} = \frac{c_{Pd} \times V_{sample\ digested}}{m_{sample\ digested}} \quad \text{(S1)}$$

where c_{Pd} is the concentration of Pd in the sample (mg/l)

$V_{sample\ digested}$ is the digested sample volume (l)

$m_{sample\ digested}$ is the total mass of digested sample (kg).

The weight percent ($wt\%_{Pd}$) of Pd in the samples was calculated by Equation (S2).

$$wt\%_{Pd} = \frac{ppm_{Pd}}{10000} \quad \text{(S2)}$$

The mass of Pd, m_{Pd}, in each sample was calculated either with Equation (S3) or Equation (S4).

$$m_{Pd} = wt\%_{Pd} \times m_{sample} \quad \text{(S3)}$$

for catalyst Cat D, Sample Exc and Sample ExS-B (where m_{sample} is the mass of the collected sample)

$$m_{Pd} = c_{Pd} \times V_{sample\ digested} \quad \text{(S4)}$$

for Samples Exa, Exb, Exd, and Exe, as well as for Samples ExS-A, ExS-C, and ExS-D.

3.3. Calculation of Measurement Errors

Two errors were taken into account. The error of the initial mass of the catalyst, as well as the error of the ICP-OES analysis. The initial error in mass was approximated to 1 mg. The ICP-OES had a measurement error of about 6%.

The total error of the extraction conversion is denoted $\Delta X_{extraction}$ and the total errors of the extraction yield, as well as of the Pd-Balance, with $\Delta Y_{extraction}$ and ΔPd-Balance.

The errors were calculated with Equations (S5) to (S17).

ΔX_{extraction}:

$$\Delta X_{extraction} = \frac{\Delta X_{extraction}}{X_{extraction}} \times X_{extraction} \qquad \text{(S5)}$$

$$\frac{\Delta X_{extraction}}{X_{extraction}} = \frac{\Delta m_{Pd,initial\,weight} - \Delta m_{Pd,final\,weight}}{m_{Pd,initial\,weight} - m_{Pd,final\,weight}} + \frac{\Delta m_{Pd,initial\,weight}}{m_{Pd,initial\,weight}} \quad \text{(S6)}$$

$$\Delta m_{Pd,initial\,weight} = \frac{1\,mg \times m_{Pd,initial\,weight}}{m_{catalyst,initial\,weight}} + 0.06 \times m_{Pd,initial\,weight} \quad \text{(S7)}$$

$$m_{Pd,final\,weight} = m_{Pd,initial\,weight} \times (1 - X_{extraction}) \quad \text{(S8)}$$

$$\Delta m_{Pd,final\,weight} = 0.06 \times m_{Pd,final\,weight} \qquad \text{(S9)}$$

ΔY_{extraction}:

$$\Delta Y_{extraction} = \frac{\Delta Y_{extraction}}{Y_{extraction}} \times Y_{extraction} \qquad \text{(S10)}$$

$$\frac{\Delta Y_{extraction}}{Y_{extraction}} = \frac{\Delta m_{Pd,initial\,weight} - \Delta m_{Pd,recovered}}{m_{Pd,initial\,weight} - m_{Pd,recovered}} + \frac{\Delta m_{Pd,initial\,weight}}{m_{Pd,initial\,weight}} \quad \text{(S11)}$$

$$m_{Pd,recovered} = m_{Pd,initial\,weight} \times Y_{extraction} \quad \text{(S12)}$$

$$\Delta m_{Pd,recovered} = 0.06 \times m_{Pd,recovered} \qquad \text{(S13)}$$

ΔPd-Balance:

$$\Delta Pd - Balance = \frac{\Delta Pd - Balance}{Pd - Balance} \times Pd - Balance \qquad \text{(S14)}$$

$$\frac{\Delta Pd - Balance}{Pd - Balance} = \frac{\Delta m_{Pd,initial\,weight} - \Delta m_{Pd,detected}}{m_{Pd,initial\,weight} - m_{Pd,detected}} + \frac{\Delta m_{Pd,initial\,weight}}{m_{Pd,initial\,weight}} \quad \text{(S15)}$$

$$m_{Pd,detected} = m_{Pd,initial\,weight} \times Pd - Balance \quad \text{(S16)}$$

$$\Delta m_{Pd,detected} = 0.06 \times m_{Pd,detected} \qquad \text{(S17)}$$

where,

$X_{extraction}$ is the conversion of Pd extraction

$Y_{extraction}$ is the yield of the Pd extraction

Pd-Balance is the Pd-Balance of the extraction experiment

$m_{Pd,initial\ weight}$ is the initial mass of Pd (m_{Pd}) for extraction (Pd content of inserted Cat D)

$\Delta m_{Pd,initial\ weight}$ is the error in determination of $m_{Pd,initial\ weight}$

$m_{Pd,final\ weight}$ is the mass of Pd remaining on the catalyst after extraction

$\Delta m_{Pd,final\ weight}$ is the error in determination of $m_{Pd,final\ weight}$

$m_{catalyst,initial\ weight}$ is the initial mass of catalyst D for extraction

$m_{Pd,recovered}$ is the mass of Pd recovered after extraction

$\Delta m_{Pd,recovered}$ is the error in determination of $m_{Pd,recovered}$

$m_{Pd,detected}$ is the mass of Pd detected after extraction anywhere in the system

$\Delta m_{Pd,detected}$ is the error in determination of $m_{Pd,detected}$

4. Supporting Experimental Data

4.1. Pd Extraction from Catalyst Cat D with Only $scCO_2$

Table S4 shows the reactant ratios used for the control experiments. Table S5 shows the Pd content measured for each sample after extraction, and for the pristine catalyst Cat D. Based on these data, along with Equations (3) – (5) (main article), the extraction conversions and extraction yields of the control experiments, as well as the Pd-Balances, were calculated. Table S6 shows the Pd distribution in the samples after the extraction. In Table S7, the extraction results are shown, as well as the corresponding measurement errors, calculated with Equations S5 – S17.

Table S4. Reactant ratios used for the control experiments (cat: catalyst; pip: piperidine; CG: Complexing group; ExS-Control: Screening experiments; Ex-Control: Detailed investigation experiments).

	Extraction System	Cat/ g	Polymer/ Pip Molar Ratio	Polymer/ Pd Molar Ratio	CG/Pd Molar Ratio	Pip/Pd Molar Ratio	$m_{Polymer}/m_{CO2}$ / wt%
E1S-Control	-	0.205	-	-	-	-	-
E2-Control	-	0.150	-	-	-	-	-
E3S-Control	pip	0.208	-	-	-	25.90	-
E4-Control	pip	0.151	-	-	-	3.78	-
E5-Control	pip	2.831	-	-	-	12	-

Table S5. Pd content of the samples from the control experiments (ExS-A to D: Samples from screening experiments; Exa to e: Samples from detailed investigation experiments).

Sample name	m_{sample} / g	$m_{sample\ digested}$ / mg	$V_{sample\ digested}$ / ml	c_{Pd} / mg/l	ppm_{Pd}	$wt\%_{Pd}$	m_{Pd} / mg
Cat D	0.20	100	100	20.083	20,083	2.0083	4.1170
E1S-A	21	21000	25	0.096	0.11	0.00001	0.0024
E1S-B	0.197	100	100	20.2	20,200	2.0200	3.9794
E1S-C	0.005	5	25	4.44	22,200	2.2200	0.1110
E1S-D	0.002	2	25	0.092	1,150	0.1150	0.0023
Cat D	0.15	100	100	20.083	20,083	2.0083	3.0125
E2a	0.1	100	25	1.28	320	0.0320	0.0320
E2b	0.001	1	25	0.124	3,100	0.3100	0.0031
E2c	0.145	101	100	18.27	18,089	1.8089	2.6229
E2d	0.001	1	25	1.17	29,250	2.9250	0.0293
E2e	0.0037	3.7	100	0.081	2,189	0.2189	0.0081
Cat D	0.21	100	100	20.083	20,083	2.0083	4.1773
E3S-A	33	33000	25	1.86	1.41	0.00014	0.0465
E3S-B	0.225	100.1	100	12.951	12,938	1.2938	2.9110
E3S-C	0.028	28	25	17.10	15,268	1.5268	0.4275
E3S-D	0.013	13	25	1.63	3,135	0.3135	0.0408
Cat D	0.151	100	100	20.083	20,083	2.0083	3.0325
E4a	0.04	40	25	0.271	169	0.0169	0.0068
E4b	0.01	10	25	0.01	25	0.0025	0.0003
E4c	0.13	100	100	17.72	17,720	1.7720	2.3036
E4d	0.03	30	25	10.55	8,792	0.8792	0.2638
E4e	0.0042	4.2	100	0.065	1,548	0.1548	0.0065
Cat D	2.831	100	100	20.083	20,083	2.0083	56.8550
E5a	0.3	300	25	0.605	50	0.0050	0.0151
E5b	0.02	20	25	0.124	155	0.0155	0.0031
E5c	2.966	100	100	15.49	15,490	1.5490	45.9433
E5d	0.04	40	25	22.2	13,875	1.3875	0.5550
E5e	0.0033	3.3	25	0.02	152	0.0152	0.0005

Table S6. Percentage of Pd in samples of control experiments (ExS-Control: Screening experiments; Ex-Control: Detailed investigation experiments; ExS-A to D: Samples from screening experiments; Exa to e: Samples from detailed investigation experiments).

	ExS-A	ExS-B	ExS-C	ExS-D	Exa	Exb	Exc	Exd	Exe	Missing
E1S-Control	0.06%	96.66%	2.70%	0.06%	-	-	-	-	-	0.52%
E2-Control	-	-	-	-	1.06%	0.10%	87.07%	0.97%	0.27%	10.53%
E3S-Control	1.11%	69.69%	10.23%	0.98%	-	-	-	-	-	17.99%
E4-Control	-	-	-	-	0.22%	0.01%	75.96%	8.70%	0.21%	14.89%
E5-Control	-	-	-	-	0.03%	0.01%	80.81%	0.98%	0.00%	18.18%

Table S7. Extraction results and errors of control experiments (ExS-Control: Screening experiments; Ex-Control: Detailed investigation experiments).

	$X_{extraction}$	$Y_{extraction}$	Pd-Balance	$\Delta X_{extraction}$	$\Delta Y_{extraction}$	ΔPd-Balance
E1S-Control	3.34%	2.81%	99.47%	0.90%	0.37%	42.24%
E2-Control	12.93%	2.41%	89.47%	2.30%	0.32%	17.00%
E3S-Control	30.32%	12.32%	82.01%	4.27%	1.61%	12.43%
E4-Control	24.04%	9.14%	85.11%	3.71%	1.22%	14.56%
E5-Control	19.19%	1.01%	81.82%	2.35%	0.12%	10.01%

4.2. Pd Extraction from Catalyst Cat D with PPh_3

Table S8 shows the reactant ratios for the PPh_3 extraction experiments. Table S9 shows the Pd content measured for each sample after extraction, and for the pristine catalyst Cat D. Based on these data, along with Equations (3) – (5), the extraction conversions and extraction yields of the experiments, and the Pd-Balances, were calculated. Table S10 shows the Pd distribution in the samples after the extraction. In Table S11, the extraction results are shown, as well as the corresponding measurement errors, calculated with Equations S5 – S17.

Table S8. Reactant ratios used for PPh_3 extraction experiments (cat: catalyst; pip: piperidine; CG: Complexing group; ExS-PPh3: Screening experiments).

	Extraction System	Cat/ g	Polymer/ Pip Molar Ratio	Polymer/ Pd Molar Ratio	CG/Pd Molar Ratio	pip/Pd Molar Ratio	$m_{Polymer}/m_{CO2}$ / wt%
E6S-PPh3	PPh_3	0.203	-	-	10.79	-	-
E7S-PPh3	PPh_3/ pip	0.204	-	-	10.34	26.41	-

Table S9. Pd content of the samples from PPh_3 extraction experiments (ExS-A to D: Samples from screening experiments).

Sample Name	m_{sample} / g	$m_{sample\ digested}$ / mg	$V_{sample\ digested}$ / ml	c_{Pd} / mg/l	ppm_{Pd}	$wt\%_{Pd}$	m_{Pd} / mg
Cat D	0.20	100	100	20.083	20,083	2.0083	4.0768
E6S-A	20	20000	25	0.061	0.08	0.00001	0.0015
E6S-B	0.181	100	100	18.8	18,800	1.8800	3.4028
E6S-C	0.021	21	25	13.10	15,595	1.5595	0.3275
E6S-D	0.004	4	25	0.041	256	0.0256	0.0010
Cat D	0.20	100	100	20.083	20,083	2.0083	4.0969
E7S-A	23	23000	25	0.75	0.82	0.0001	0.0188
E7S-B	0.194	100.4	100	16.20	16,135	1.6135	3.1303
E7S-C	0.024	24	25	13.2	13,750	1.3750	0.3300
E7S-D	0.015	15	25	2.01	3,350	0.3350	0.0503

Table S10. Percentage of Pd in samples for PPh_3 extraction experiments (ExS-PPh3: Screening experiments; ExS-A to D: Samples from screening experiments; Exa to e: Samples from detailed investigation experiments).

	ExS-A	ExS-B	ExS-C	ExS-D	Exa	Exb	Exc	Exd	Exe	Missing
E6S-PPh3	0.04%	83.47%	8.03%	0.03%	-	-	-	-	-	8.43%
E7S-PPh3	0.46%	76.41%	8.05%	1.23%	-	-	-	-	-	13.85%

Table S11. Extraction results and errors of PPh_3 extraction experiments (ExS-PPh3: Screening experiments).

	$X_{extraction}$	$Y_{extraction}$	Pd-Balance	$\Delta X_{extraction}$	$\Delta Y_{extraction}$	ΔPd-Balance
E6S-PPh3	16.53%	8.10%	91.56%	2.56%	1.05%	16.78%
E7S-PPh3	23.59%	9.74%	86.15%	3.44%	1.27%	13.81%

4.3. Pd Extraction from Catalyst Cat D with Polymer p(FDA)SH

Table S12 shows the reactant ratios used in the screening experiments for p(FDA)SH extraction experiments. Table S13 shows the Pd content measured for each sample after extraction and for the pristine catalyst Cat D. Based on these data, along with Equations (3) – (5), the conversions and extraction yields of the experiments, as well as the Pd-Balances, were calculated. Table S14 shows the Pd distribution in the samples after the extraction. In Table S15, the extraction results are shown, as well as the corresponding measurement errors, calculated with Equations S5 – S17.

Table S12. Reactant ratios used for extraction experiments with p(FDA)SH (cat: catalyst; pip: piperidine; CG: Complexing group; ExS-p(FDA)SH: Screening experiments).

	Extraction System	Cat/ g	Polymer/ Pip Molar Ratio	Polymer/ Pd Molar Ratio	CG/Pd Molar Ratio	pip/Pd Molar Ratio	$m_{Polymer}/m_{CO2}$ / wt%
E8S-p(FDA)SH	$p(FDA)_{11}SH$	0.202	-	10.34	10.34	-	6.55
E9S-p(FDA)SH	$p(FDA)_{11}SH$/ pip	0.204	0.39	10.21	10.21	26.41	6.56

Table S13. Pd content of the samples from extraction experiments with p(FDA)SH (ExS-A to D: Samples from screening experiments).

Sample Name	m_{sample} / g	$m_{sample\ digested}$ / mg	$V_{sample\ digested}$ / ml	c_{Pd} / mg/l	ppm_{Pd}	$wt\%_{Pd}$	m_{Pd} / mg
Cat D	0.20	100	100	20.083	20,083	2.0083	4.0577
E8S-A	26	26000	25	0.124	0.12	0.00001	0.0031
E8S-B	0.231	100	100	14.4	14,400	1.4400	3.3264
E8S-C	0.014	14	25	4.53	8,089	0.8089	0.1133
E8S-D	0.01	10	25	0.010	25	0.0025	0.0003
Cat D	0.20	100	100	20.083	20,083	2.0083	4.0969
E9S-A	29	29000	25	1.93	1.66	0.0002	0.0483
E9S-B	0.272	100	100	12.30	12,300	1.2300	3.3456
E9S-C	0.032	32	25	11.1	8,672	0.8672	0.2775
E9S-D	0.005	5	25	0.18	900	0.0900	0.0045

Table S14. Percentage of Pd in the samples for extraction experiments with p(FDA)SH (ExS-p(FDA)SH: Screening experiments; ExS-A to D: Samples from screening experiments; Exa to e: Samples from detailed investigation experiments).

	ExS-A	ExS-B	ExS-C	ExS-D	Exa	Exb	Exc	Exd	Exe	Missing
E8S-p(FDA)SH	0.08%	81.98%	2.79%	0.01%	-	-	-	-	-	15.14%
E9S-p(FDA)SH	1.18%	81.66%	6.77%	0.11%	-	-	-	-	-	10.28%

Table S15. Extraction results and errors of p(FDA)SH extraction experiments (ExS-p(FDA)SH: Screening experiments).

	$X_{extraction}$	$Y_{extraction}$	Pd-Balance	$\Delta X_{extraction}$	$\Delta Y_{extraction}$	ΔPd-Balance
E8S-p(FDA)SH	18.02%	2.87%	84.85%	2.75%	0.37%	13.37%
E9S-p(FDA)SH	18.34%	8.06%	89.72%	2.78%	1.05%	15.49%

4.4. *Pd Extraction from Catalyst Cat D with Polymer p(FDA-co-DPPS)*

Table S16 shows the reactant ratios used for p(FDA-*co*-DPPS) extraction tests at standard conditions (40 °C, 25 MPa). Table S17 shows the Pd content measured for each sample after extraction and for the pristine catalyst Cat D. Based on these data, along with Equations (3) – (5), the extraction conversions and extraction yields of the experiments, as well as the Pd-Balances, were calculated. Table S18 shows the Pd distribution in the samples after the extraction. In Table S19, the extraction results are shown, as well as the corresponding measurement errors, calculated with Equations S5 – S17.

Table S16. Reactant ratios used for extraction experiments with p(FDA-*co*-DPPS) (40 °C, 25 MPa) (cat: catalyst; pip: piperidine; CG: Complexing group; ExS-DPPS: Screening experiments; Ex-DPPS: Detailed investigation experiments).

	**Extraction System**	**Cat/ g**	**Polymer/ Pip Molar Ratio**	**Polymer/ Pd Molar Ratio**	**CG/Pd Molar Ratio**	**pip/Pd Molar Ratio**	**$m_{Polymer}$/ m_{CO2} / wt%**
E10S-DPPS	p(FDA_{18}-*co*-$DPPS_7$)	0.203	-	2.09	14.60	-	2.75
E11S-DPPS	p(FDA_{18}-*co*-$DPPS_7$)/ pip	0.201	0.40	2.15	17.18	5.36	2.82
E12-DPPS	p(FDA_{18}-*co*-$DPPS_7$)/ pip	0.162	0.39	1.49	12	3.81	0.25
E13-DPPS	p(FDA_{18}-*co*-$DPPS_7$)/ pip	0.160	0.39	1.49	12	3.81	0.25
E14-DPPS	p(FDA_{26}-*co*-$DPPS_{10}$)/ pip	0.328	0.20	1.09	12	5.45	0.51
E15-DPPS	p(FDA_{26}-*co*-$DPPS_{10}$)/ pip	0.321	0.20	1.09	12	5.45	0.50

Table S17. Pd content of the samples from the extraction experiments with p(FDA-*co*-DPPS) (40 °C, 25 MPa) (ExS-A to D: Samples from screening experiments; Exa to e: Samples from detailed investigation experiments).

Sample Name	m_{sample} / g	$m_{sample\ digested}$ / mg	$V_{sample\ digested}$ / ml	c_{Pd} / mg/l	ppm_{Pd}	$wt\%_{Pd}$	m_{Pd} / mg
Cat D	0.20	100	100	20.083	20,083	2.0083	4.0768
E10S-A	128	128000	25	1.950	0.38	0.00004	0.0488
E10S-B	0.48	200	100	11.6	5,800	0.5800	2.7840
E10S-C	0.12	120	25	11.20	2,333	0.2333	0.2800
E10S-D	Sample not collected						
Cat D	0.20	100	100	20.083	20,083	2.0083	4.0367
E11S-A	134	134000	25	1.00	0.19	0.00002	0.0249
E11S-B	0.123	100.6	100	11.90	11,829	1.1829	1.4550
E11S-C	0.036	36	25	14.9	10,347	1.0347	0.3725
E11S-D	Sample not collected						
Cat D	0.162	100	100	20.083	20,083	2.0083	3.2534
E12a	0.6	600	250	0.055	23	0.0023	0.0138
E12b	0.03	30	25	0.14	117	0.0117	0.0035
E12c	0.102	97	25	49.1	12,655	1.2655	1.2908
E12d	0.11	110	25	18.5	4,205	0.4205	0.4625
E12e	0.0035	3.5	25	0.566	4,043	0.4043	0.0142
Cat D	0.16	100	100	20.083	20,083	2.0083	3.2133
E13a	0.09	90	25	0.318	88	0.0088	0.0080
E13b	0.01	10	25	0.01	25	0.0025	0.0003
E13c	0.196	101	100	7.94	7,861	0.7861	1.5408
E13d	0.21	210	25	30.1	3,583	0.3583	0.7525
E13e	0.0032	3.2	100	0.635	19,844	1.9844	0.0635
Cat D	0.328	100	100	20.083	20,083	2.0083	6.5872
E14a	0.74	740	25	1.05	35	0.0035	0.0263
E14b	0.04	40	25	0.01	6	0.0006	0.0003
E14c	0.273	101	100	9.18	9,089	0.9089	2.4813
E14d	0.29	290	25	80.2	6,914	0.6914	2.0050
E14e	0.0033	3.3	25	0.041	311	0.0311	0.0010
Cat D	0.321	100	100	20.083	20,083	2.0083	6.4466
E15a	0.48	480	25	0.293	15	0.0015	0.0073
E15b	0.1	100	25	0.01	2	0.0002	0.0003
E15c	0.245	100	100	10.1	10,100	1.0100	2.4745
E15d	0.25	250	25	77.8	7,780	0.7780	1.9450
E15e	0.003	3	25	0.02	167	0.0167	0.0005

Table S18. Percentage of Pd in the samples of the extraction experiments with p(FDA-*co*-DPPS) (40 °C, 25 MPa) (ExS-DPPS: Screening experiments; Ex-DPPS: Detailed investigation experiments; ExS-A to D: Samples from screening experiments; Exa to e: Samples from detailed investigation experiments).

	ExS-A	ExS-B	ExS-C	ExS-D	Exa	Exb	Exc	Exd	Exe	Missing
E10S-DPPS	1.20%	68.29%	6.87%	-	-	-	-	-	-	23.64%
E11S-DPPS	0.62%	36.04%	9.23%	-	-	-	-	-	-	54.11%
E12-DPPS	-	-	-	-	0.42%	0.11%	39.67%	14.22%	0.43%	45.15%
E13-DPPS	-	-	-	-	0.25%	0.01%	47.95%	23.42%	1.98%	26.40%
E14-DPPS	-	-	-	-	0.40%	0.00%	37.67%	30.44%	0.02%	31.48%
E15-DPPS	-	-	-	-	0.11%	0.00%	38.38%	30.17%	0.01%	31.32%

Table S19. Extraction results and errors of the extraction experiments with p(FDA-*co*-DPPS) (40 °C, 25 MPa) (ExS-DPPS: Screening experiments; Ex-DPPS: Detailed investigation experiments).

	$X_{extraction}$	$Y_{extraction}$	Pd-Balance	$\Delta X_{extraction}$	$\Delta Y_{extraction}$	ΔPd-Balance
E10S-DPPS	31.71%	8.06%	76.35%	4.45%	1.05%	11.13%
E11S-DPPS	63.96%	9.84%	45.89%	8.49%	1.28%	6.16%
E12-DPPS	60.33%	15.18%	54.85%	8.23%	2.03%	7.67%
E13-DPPS	52.05%	25.65%	73.60%	7.20%	3.45%	11.03%
E14-DPPS	62.33%	30.86%	68.52%	7.97%	3.93%	9.10%
E15-DPPS	61.62%	30.30%	68.68%	7.90%	3.87%	9.14%

Parameter Screening:

Table S20 shows the reactant ratios used for p(FDA-*co*-DPPS) extraction tests at parameter screening. Table S21 shows the Pd content measured for each sample after extraction and for the pristine catalyst Cat D. Based on these data, along with Equations (3) – (5), the extraction conversions and extraction yields of the experiments, as well as the Pd-Balances, were calculated. Table S22 shows the Pd distribution in the samples after the extraction. In Table S23, the extraction results are shown, as well as the corresponding measurement errors, calculated with Equations S5 – S17.

Table S20. Reactant ratios used for extraction experiments with p(FDA-*co*-DPPS) at parameter screening (cat: catalyst; pip: piperidine; CG: Complexing group; Ex-DPPS: Detailed investigation experiments).

	**Extraction System**	**Cat/ g**	**Polymer/ Pip Molar Ratio**	**Polymer/ Pd Molar Ratio**	**CG/Pd Molar Ratio**	**pip/Pd Molar Ratio**	**$m_{Polymer}/m_{CO2}$ / wt%**
E12-DPPS	p(FDA$_{18}$-*co*-DPPS$_7$)/ pip	0.162	0.39	1.49	12	3.81	0.25
E13-DPPS	p(FDA$_{18}$-*co*-DPPS$_7$)/ pip	0.160	0.39	1.49	12	3.81	0.25
E14-DPPS	p(FDA$_{26}$-*co*-DPPS$_{10}$)/ pip	0.328	0.20	1.09	12	5.45	0.51
E15-DPPS	p(FDA$_{26}$-*co*-DPPS$_{10}$)/ pip	0.321	0.20	1.09	12	5.45	0.50
E16-DPPS	p(FDA$_{26}$-*co*-DPPS$_{10}$)/ pip	0.092	0.20	3.81	42	19.09	0.50
E17-DPPS	p(FDA$_{26}$-*co*-DPPS$_{10}$)/ pip	0.094	0.20	3.81	42	19.09	0.50
E18-DPPS	p(FDA$_{26}$-*co*-DPPS$_{10}$)/ pip	0.765	0.20	0.45	5	2.27	0.50
E19-DPPS	p(FDA$_{18}$-*co*-DPPS$_7$)/ pip	0.170	0.31	1.5	12	4.77	0.24
E20-DPPS	p(FDA$_{26}$-*co*-DPPS$_{10}$)/ pip	0.321	0.20	1.09	12	5.45	0.50
E21-DPPS	p(FDA$_{26}$-*co*-DPPS$_{10}$)/ pip	0.323	0.20	1.09	12	5.45	0.50
E22-DPPS	p(FDA$_{26}$-*co*-DPPS$_{10}$)/ pip	0.320	0.20	1.09	12	5.45	0.57
E23-DPPS	p(FDA$_{26}$-*co*-DPPS$_{10}$)/ pip	0.324	0.20	1.09	12	5.45	0.54

Table S21. Pd content of the samples from the extraction experiments with p(FDA-*co*-DPPS) at parameter screening (Exa to e: Samples from detailed investigation experiments).

Sample Name	m_{sample} / g	$m_{sample\ digested}$ / mg	$V_{sample\ digested}$ / ml	c_{Pd} / mg/l	ppm_{Pd}	$wt\%_{Pd}$	m_{Pd} / mg
Cat D	0.162	100	100	20.083	20,083	2.0083	3.2534
E12a	0.6	600	250	0.055	23	0.0023	0.0138
E12b	0.03	30	25	0.14	117	0.0117	0.0035
E12c	0.102	97	25	49.1	12,655	1.2655	1.2908
E12d	0.11	110	25	18.5	4,205	0.4205	0.4625
E12e	0.0035	3.5	25	0.566	4,043	0.4043	0.0142
Cat D	0.16	100	100	20.083	20,083	2.0083	3.2133
E13a	0.09	90	25	0.318	88	0.0088	0.0080
E13b	0.01	10	25	0.01	25	0.0025	0.0003
E13c	0.196	101	100	7.94	7,861	0.7861	1.5408
E13d	0.21	210	25	30.1	3,583	0.3583	0.7525
E13e	0.0032	3.2	100	0.635	19,844	1.9844	0.0635
Cat D	0.328	100	100	20.083	20,083	2.0083	6.5872
E14a	0.74	740	25	1.05	35	0.0035	0.0263
E14b	0.04	40	25	0.01	6	0.0006	0.0003
E14c	0.273	101	100	9.18	9,089	0.9089	2.4813
E14d	0.29	290	25	80.2	6,914	0.6914	2.0050
E14e	0.0033	3.3	25	0.041	311	0.0311	0.0010
Cat D	0.321	100	100	20.083	20,083	2.0083	6.4466
E15a	0.48	480	25	0.293	15	0.0015	0.0073
E15b	0.1	100	25	0.01	2	0.0002	0.0003
E15c	0.245	100	100	10.1	10,100	1.0100	2.4745
E15d	0.25	250	25	77.8	7,780	0.7780	1.9450
E15e	0.003	3	25	0.02	167	0.0167	0.0005
Cat D	0.092	100	100	20.083	20,083	2.0083	1.8476
E16a	0.67	670	25	0.104	4	0.0004	0.0026
E16b	0.1	100	25	0.022	6	0.0006	0.0006
E16c	0.169	100	100	7.03	7,030	0.7030	1.1881
E16d	0.11	110	25	21.96	4,991	0.4991	0.5490
E16e	0.0036	3.6	25	0.02	139	0.0139	0.0005
Cat D	0.094	100	100	20.083	20,083	2.0083	1.8878
E17a	0.46	460	25	0.115	6	0.0006	0.0029
E17b	0.17	170	25	0.289	43	0.0043	0.0072
E17c	0.104	100	100	11.82	11,820	1.1820	1.2293
E17d	0.03	30	25	10.23	8,525	0.8525	0.2558
E17e	0.0029	2.9	25	0.05	431	0.0431	0.0013
Cat D	0.765	100	100	20.083	20,083	2.0083	15.3635
E18a	0.9	900	25	0.172	5	0.0005	0.0043
E18b	0.08	80	25	0.01	3	0.0003	0.0003
E18c	0.977	100	100	13.6	13,600	1.3600	13.2872
E18d	0.01	10	25	7.2	18,000	1.8000	0.1800
E18e	0.0032	3.2	25	0.02	156	0.0156	0.0005

Table S21 (continuated).

Sample Name	m_{sample} / g	$m_{sample\ digested}$ / mg	$V_{sample\ digested}$ / ml	c_{Pd} / mg/l	ppm_{Pd}	$wt\%_{Pd}$	m_{Pd} / mg
Cat D	0.17	100	100	20.083	20,083	2.0083	3.4141
E19a	0.36	360	25	0.728	51	0.0051	0.0182
E19b	0.03	30	25	0.017	14	0.0014	0.0004
E19c	0.215	100	100	14.67	14,670	1.4670	3.1541
E19d	0.01	10	25	2.28	5,700	0.5700	0.0570
E19e	0.003	3	25	0.028	233	0.0233	0.0007
Cat D	0.321	100	100	20.083	20,083	2.0083	6.4466
E20a	1.2	1200	25	0.875	18	0.0018	0.0219
E20b	0.07	70	25	0.151	54	0.0054	0.0038
E20c	0.394	116	100	16.8	14,483	1.4483	5.7062
E20d	0.1	100	25	23.52	5,880	0.5880	0.5880
E20e	0.0033	3.3	25	0.01	76	0.0076	0.0003
Cat D	0.323	100	100	20.083	20,083	2.0083	6.4868
E21a	1.03	1030	25	0.767	19	0.0019	0.0192
E21b	0.04	40	25	0.101	63	0.0063	0.0025
E21c	0.405	114	100	16.9	14,825	1.4825	6.0039
E21d	0.05	50	25	11	5,500	0.5500	0.2750
E21e	0.0035	3.5	25	0.014	100	0.0100	0.0004
Cat D	0.32	100	100	20.083	20,083	2.0083	6.4266
E22a	0.5	500	25	0.37	19	0.0019	0.0093
E22b	0.17	170	25	0.777	114	0.0114	0.0194
E22c	0.434	100	100	13.53	13,530	1.3530	5.8720
E22d	0.04	40	25	16.9	10,563	1.0563	0.4225
E22e	0.003	3	25	0.02	167	0.0167	0.0005
Cat D	0.324	100	100	20.083	20,083	2.0083	6.5069
E23a	0.48	480	25	0.344	18	0.0018	0.0086
E23b	0.15	150	25	0.295	49	0.0049	0.0074
E23c	0.478	108	100	12.3	11,389	1.1389	5.4439
E23d	0.13	130	25	32.2	6,192	0.6192	0.8050
E23e	0.0029	2.9	25	0.053	457	0.0457	0.0013

Table S22. Percentage of Pd in the samples of the extraction experiments with p(FDA-*co*-DPPS) at parameter screening (Ex-DPPS: Detailed investigation experiments; ExS-A to D: Samples from screening experiments; Exa to e: Samples from detailed investigation experiments).

	**ExS-A**	**ExS-B**	**ExS-C**	**ExS-D**	**Exa**	**Exb**	**Exc**	**Exd**	**Exe**	**Missing**
E12-DPPS	-	-	-	-	0.42%	0.11%	39.67%	14.22%	0.43%	45.15%
E13-DPPS	-	-	-	-	0.25%	0.01%	47.95%	23.42%	1.98%	26.40%
E14-DPPS	-	-	-	-	0.40%	0.00%	37.67%	30.44%	0.02%	31.48%
E15-DPPS	-	-	-	-	0.11%	0.00%	38.38%	30.17%	0.01%	31.32%
E16-DPPS	-	-	-	-	0.14%	0.03%	64.30%	29.71%	0.03%	5.79%
E17-DPPS	-	-	-	-	0.15%	0.38%	65.12%	13.55%	0.07%	20.73%
E18-DPPS	-	-	-	-	0.03%	0.00%	86.49%	1.17%	0.00%	12.31%
E19-DPPS	-	-	-	-	0.53%	0.01%	92.38%	1.67%	0.02%	5.38%
E20-DPPS	-	-	-	-	0.34%	0.06%	88.51%	9.12%	0.00%	1.96%
E21-DPPS	-	-	-	-	0.30%	0.04%	92.56%	4.24%	0.01%	2.86%
E22-DPPS	-	-	-	-	0.14%	0.30%	91.37%	6.57%	0.01%	1.60%
E23-DPPS	-	-	-	-	0.13%	0.11%	83.66%	12.37%	0.02%	3.70%

Table S23. Extraction results and errors of the extraction experiments with p(FDA-*co*-DPPS) at parameter screening (Ex-DPPS: Detailed investigation experiments).

	$X_{extraction}$	$Y_{extraction}$	Pd-Balance	$\Delta X_{extraction}$	$\Delta Y_{extraction}$	ΔPd-Balance
E12-DPPS	60.33%	15.18%	54.85%	8.23%	2.03%	7.67%
E13-DPPS	52.05%	25.65%	73.60%	7.20%	3.45%	11.03%
E14-DPPS	62.33%	30.86%	68.52%	7.97%	3.93%	9.10%
E15-DPPS	61.62%	30.30%	68.68%	7.90%	3.87%	9.14%
E16-DPPS	35.70%	29.91%	94.21%	5.76%	4.38%	30.03%
E17-DPPS	34.88%	14.15%	79.27%	5.62%	2.02%	14.42%
E18-DPPS	13.51%	1.20%	87.69%	1.77%	0.15%	11.57%
E19-DPPS	7.62%	2.24%	94.62%	1.55%	0.29%	22.25%
E20-DPPS	11.49%	9.52%	98.04%	1.73%	1.21%	27.63%
E21-DPPS	7.44%	4.58%	97.14%	1.23%	0.58%	22.46%
E22-DPPS	8.63%	7.03%	98.40%	1.37%	0.89%	31.33%
E23-DPPS	16.34%	12.64%	96.30%	2.32%	1.60%	19.89%

References

[1] Severac, R.; Lacroix-Desmazes, P.; Boutevin, B. Reversible addition-fragmentation chain-transfer (RAFT) copolymerization of vinylidene chloride and methyl acrylate. *Polym. Int.* **2002**, *51*, 1117 – 1122. https://doi.org/10.1002/pi.932.

[2] Ruiu, A.; Bauer-Siebenlist, B.; Senila, M.; Jänisch, T.; Foix, D.; Seaudeau-Pirouley, K.; Lacroix-Desmazes, P. Promising polymer-assisted extraction of palladium from supported catalysts in supercritical carbon dioxide. *Journal of CO_2 Utilization* **2020**, *41*, 101232–101238. https://doi.org/10.1016/j.jcou.2020.101232.

[3] Otto, K.; Haack, L. P.; deVries, J. E. Identification of two types of oxidized palladium on γ-alumina by X-ray photoelectron spectroscopy. *Appl. Catal. B, Environ.* **1992**, *1*, 1–12. https://doi.org/10.1016/0926-3373(92)80003-I.

[4] Ribaut, T.; Oberdisse, J.; Annighofer, B.; Fournel, B.; Sarrade, S.; Haller, H.; Lacroix-Desmazes, P. Solubility and Self-Assembly of Amphiphilic Gradient and Block Copolymers in Supercritical CO_2. J. *Phys. Chem. B.* **2011**, *115*, 836–843. https://doi.org/10.1021/jp108888x.

[5] Ribaut, T.; Oberdisse, J.; Annighofer, B.; Stoychev, I.; Fournel, B.; Sarrade, S.; Lacroix-Desmazes, P. SANS study of the self-organization of gradient copolymers with ligand groups in supercritical CO_2. *Soft Matter* **2009**, *5*, 4962–4970. https://doi.org/10.1039/b912268k.
